# Roles for the long non-coding RNA *Pax6os1*/*PAX6-AS1* in pancreatic beta cell function

**DOI:** 10.1016/j.isci.2024.111518

**Published:** 2024-12-09

**Authors:** Livia Lopez-Noriega, Rebecca Callingham, Aida Martinez-Sánchez, Sameena Nawaz, Grazia Pizza, Nejc Haberman, Nevena Cvetesic, Marie-Sophie Nguyen-Tu, Boris Lenhard, Piero Marchetti, Lorenzo Piemonti, Eelco de Koning, A.M. James Shapiro, Paul R. Johnson, Isabelle Leclerc, Benoit Hastoy, Benoit R. Gauthier, Timothy J. Pullen, Guy A. Rutter

**Affiliations:** 1 Section of Cell Biology and Functional Genomics, Department of Medicine, Endocrinology and Metabolism, Imperial College London, London, UK; 2 Oxford Centre for Diabetes, Endocrinology and Metabolism (OCDEM), Radcliffe Department of Medicine, University of Oxford, Oxford, UK; 3 Computational Regulatory Genomics, MRC Laboratory of Medical Sciences, London, UK; 4 Institute of Clinical Sciences, Faculty of Medicine, Imperial College London, London, UK; 5 Department of Clinical and Experimental Medicine, University of Pisa, Pisa, Italy; 6 San Raffaele Diabetes Research Institute (SR–DRI), Istituto di Ricovero e Cura a Carattere Scientifico (IRCCS) San Raffaele Scientific Institute, Milan, Italy; 7 Vita-Salute San Raffaele University, Milan, Italy; 8 Department of Medicine, Leiden University Medical Center, Leiden, the Netherlands; 9 Hubrecht Institute, Utrecht, the Netherlands; 10 Clinical Islet Laboratory and Clinical Islet Transplant Program, University of Alberta, Edmonton, AB, Canada; 11 Nuffield Department of Surgical Sciences, University of Oxford, Oxford, UK; 12 Andalusian Center of Molecular Biology and Regenerative Medicine CABIMER, Junta de Andalucia-University of Pablo de Olavide-University of Seville-CSIC, Seville, Spain; 13 Centro de Investigacion Biomedica en Red de Diabetes y Enfermedades Metabolicas Asociadas (CIBERDEM), Madrid, Spain; 14 Department of Diabetes, King’s College London, London, UK; 15 Lee Kong Chian School of Medicine, Nanyang Technological University, Singapore, Singapore; 16 CR-CHUM, Université de Montréal, Montréal, QC, Canada; 17 Research Institute of McGill University Health Centre, Montréal, QC, Canada

**Keywords:** Cell biology, Cellular physiology, Molecular biology

## Abstract

Long non-coding RNAs (lncRNAs) are emerging as crucial regulators of beta cell function. Here, we show that an lncRNA-transcribed antisense to Pax6, annotated as Pax6os1/PAX6-AS1, was upregulated by high glucose concentrations in human as well as murine beta cell lines and islets. Elevated expression was also observed in islets from mice on a high-fat diet and patients with type 2 diabetes. Silencing *Pax6os1*/*PAX6-AS1* in MIN6 or EndoC-βH1 cells increased several beta cell signature genes’ expression. Pax6os1/PAX6-AS1 was shown to bind to EIF3D, indicating a role in translation of specific mRNAs, as well as histones H3 and H4, suggesting a role in histone modifications. Important interspecies differences were found, with a stronger phenotype in humans. Only female *Pax6os1* null mice fed a high-fat diet showed slightly enhanced glucose clearance. In contrast, silencing *PAX6-AS1* in human islets enhanced glucose-stimulated insulin secretion and increased calcium dynamics, whereas overexpression of the lncRNA resulted in the opposite phenotype.

## Highlights

• Glucose upregulates *Pax6os1/PAX6-AS1* in human and mouse beta cell lines and islets
• Silencing *Pax6os1*/*PAX6-AS1* increases beta cell signature gene expression
• On a high-fat diet, female *Pax6os1* null mice show slightly enhanced glucose tolerance
• *Pax6os1*/*PAX6-AS1* bind EIF3D and histones H3 and H4, suggesting a role in epigenetic regulation

## Graphical abstract

**Figure:**
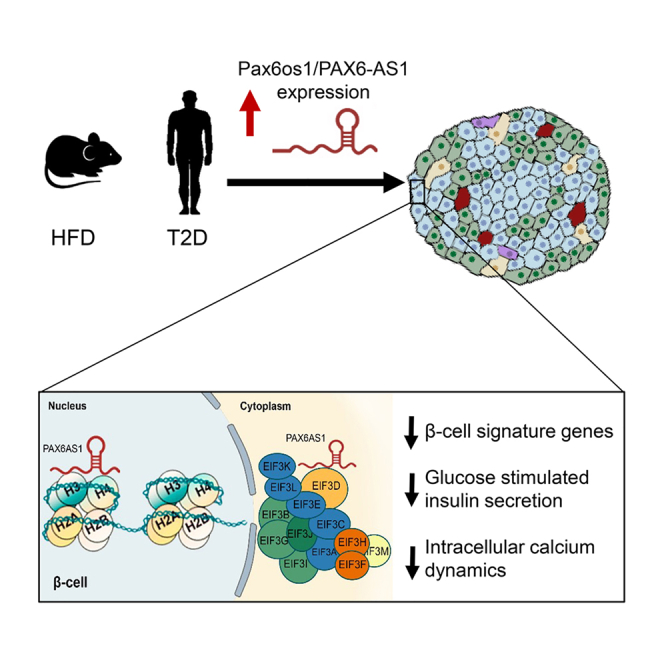

## Introduction

Type 2 diabetes (T2D) develops when beta cells within pancreatic islets no longer secrete sufficient insulin to lower circulating blood glucose levels, usually in the presence of insulin resistance.^1^ However, in a subset of T2D patients, defective insulin secretion is observed despite near-normal insulin sensitivity.^2^ Therefore, in all forms of the disease, changes in beta cell “identity” are thought to play an important role in functional impairment and the selective loss of glucose responsiveness.^3^

Loss of normal beta cell function is often characterized by decreased expression of insulin (*INS*) and of genes critical for glucose entry and metabolism.^3^^,^^4^ These changes may be accompanied by increased expression of so-called “disallowed genes,” whose levels are unusually low in healthy beta cells compared to other cell types.^5^ Furthermore, in several models of diabetes, the aforementioned changes are associated with decreased expression of transcription factors that are required to maintain a mature beta cell phenotype, including pancreatic duodenum homeobox-1 (*PDX1*)^6^ and MAF BZIP transcription factor A (*MAFA*).^6^^,^^7^ The transcription factor Pax6 regulates the expression of several genes involved in insulin processing and secretion, while repressing signature genes defining different endocrine cell lineages, such as ghrelin (Ghrl).^8^^,^^9^^,^^10^ As a result, Pax6 expression is key to maintaining beta cell identity and function. Embryonic deletion of Pax6 in the murine pancreas leads to a drastic reduction in the number of alpha and beta cells, resulting in the death of mutant mice at postnatal days 3–6 due to severe hyperglycaemia.^11^ Conditional inactivation of Pax6 in adult mice leads to impaired beta cell function and glucose intolerance,^12^ demonstrating the continued importance of this gene in mature beta cells. Further highlighting the importance of this locus in diabetes, pancreatic Pax6 *cis*-regulatory elements that interact with the Pax6 promoter and neighboring long non-coding RNAs modulate the activity of pancreas-related transcription factors such as Pax4.^13^ In humans, loss-of-function mutations in *PAX6* are associated with aniridia (iris hypoplasia) and T2D.^14^^,^^15^

Long non-coding RNAs (lncRNAs), defined as transcripts >200 nucleotides in length that are not translated into proteins, are crucial components of the pancreatic islet regulome, whose misexpression may contribute to the development of T2D.^16^ LncRNAs are expressed in a tissue-/cell-specific manner and more than 1,100 have been identified in human and murine pancreatic islets.^17^^,^^18^ Furthermore, the expression of several of these is modulated by high glucose concentrations, suggesting that they may be involved in beta cell compensation in response to high insulin demand.^19^ Interestingly, a number of beta-cell-enriched lncRNAs are mapped to genetic loci in the proximity of beta cell signature genes, such as *PLUTO*-*PDX1* or *Paupar-Pax6*, and regulate their expression in *cis.*^19^^,^^20^

In the current study, we sought to determine whether a lncRNA expressed from the *PAX6* locus, previously annotated as *Pax6 opposite strand 1* (*Pax6os1*) in mice and *PAX6 antisense 1* (*PAX6-AS1*) in humans,^21^ might impact beta cell identity and/or function through the modulation of *Pax6* expression or by other mechanisms.

## Results

### *Pax6os1*/*PAX6-AS1* expression is enriched in pancreatic islets and upregulated by high glucose as well as in T2D

**Figure 1:**
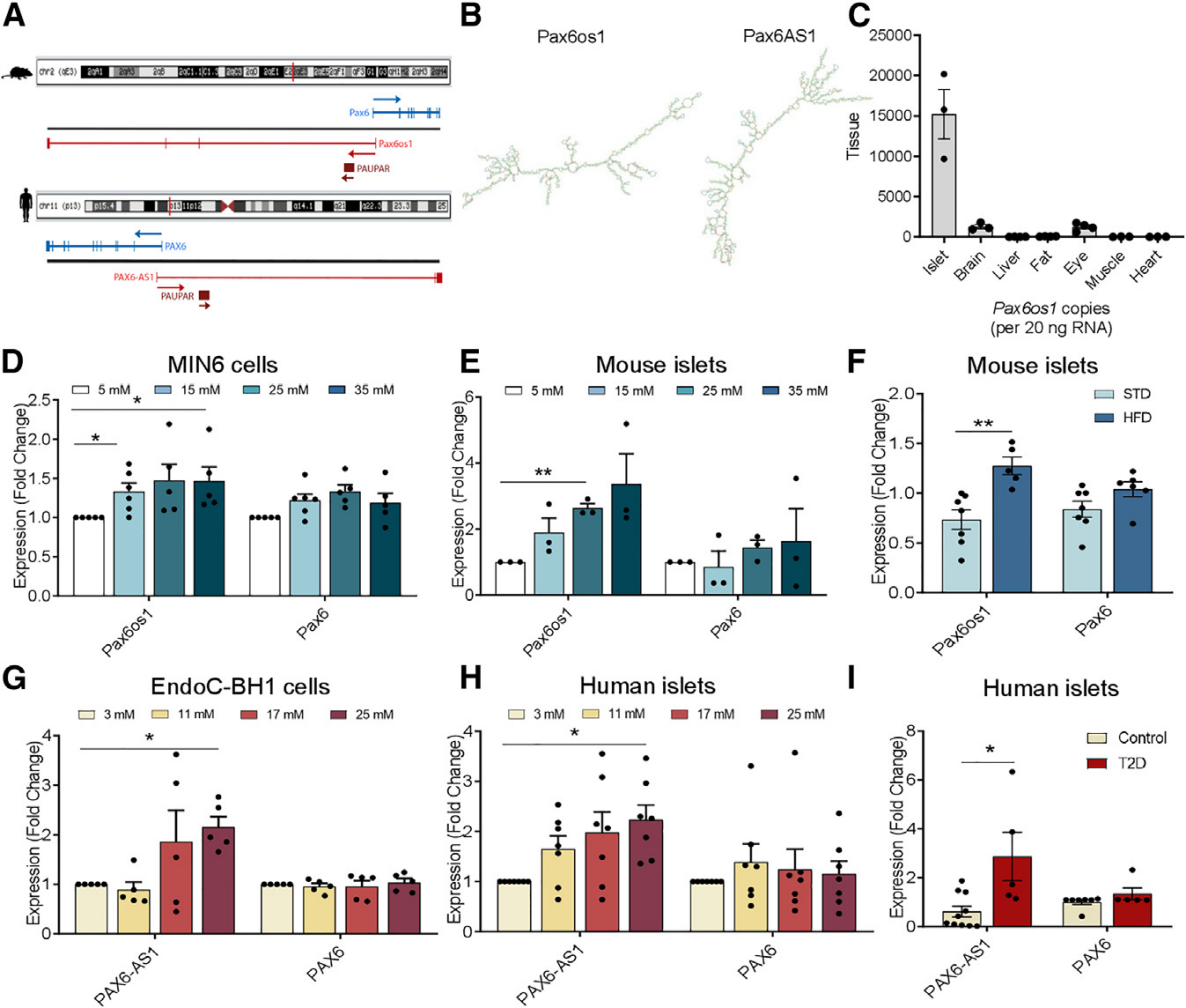
*Pax6os1* is chiefly expressed in pancreatic islets in the mouse and is upregulated by high-fat diet as well as in islets from patients with type 2 diabetes (A) Schematic representation of the long non-coding RNA identified at the Pax6 locus in mice and humans. (B) Secondary structure predicted using RNAfold from the VIENA package for Pax6os1 and PAX6-AS1, respectively, and drew using FORNA webserver. (C) Tissue distribution of *Pax6os1* expression. *n* = 3. (D and E) *Pax6os1* and Pax6 expression in MIN6 cells and CD1 mouse pancreatic islets cultured at different glucose concentrations for 48 h (note that the standard glucose concentration for MIN6 cells culture is 25 mM). MIN6cells: *n* = 6. CD1 islets: *n* = 3. (F) *Pax6os1* and Pax6 expression in pancreatic islets from C57/BL6 mice in standard (STD) or high-fat diet (HFD) for 8 weeks. *n* = 6. (G and H) *PAX6-AS1* and PAX6 mRNA expression in EndoC-βH1 cells and human islets cultured with different glucose concentrations for 48 h (note that the standard concentration for EndoC-βH1 culture is 5.5 mM). EndoC-βH1: *n* = 5. Human islets: *n* = 7. (I) *PAX6-AS1* and PAX6 expression in human pancreatic islets from normoglycemic or diabetic donors. Control: *n* = 10; Diabetic: *n* = 5. Data are represented as the mean ± SEM. ∗*p* < 0.05; one-way ANOVA repeated measurements.

The lncRNA *Pax6os1*/*PAX6-AS1* is a 1,464/1,656 nucleotide transcript mapped to a syntenically conserved region in chromosome 2 in mice and chromosome 11 in humans. It is transcribed antisense to the *Pax6* gene, overlapping with intron 1 in both species. The first intron of *Pax6os1*/*PAX6-AS1* also overlaps with *Paupar*, another lncRNA that is mainly expressed in alpha cells, and it is involved in Pax6 splicing.^17^ As opposed to other lncRNAs including *Paupar*, *Pax6os1/PAX6-AS1* is not highly conserved at the nucleotide level between species, containing four exons in mice and three in humans (Figure 1A), whereas important differences are also found in the predicted secondary structures (Figure 1B). A strong tendency toward moderate correlation between Pax6os1 and PAX6AS1 was found in 2mers (r = 0.469), whereas 3mers (r = 0.198), 4mers (r = 0.92), and 5mers (r = 0.03) only showed weak correlations far from being statistically significant (Figures S1A–S1C). Curiously, a correlation was also found in 2mers between PAX6-AS1 and MALAT1 (r = 0.61) (Figure S1 related to Figure 1), an lncRNA that has been shown to be located both in the nucleus and cytoplasm in beta cells, where it is involved in chromatin remodeling and microRNA sponging, respectively.^16^ Tissue distribution of *Pax6os1* in the mouse is similar to that described for *Pax6*,^22^ being predominantly expressed in pancreatic islets and, to a lesser extent, in the eye and brain (Figure 1C). Within the islet, previously published data indicate that *Pax6os1* is enriched in beta and delta cells,^23^ whereas there is no detectable expression in alpha cells, where *Paupar* is strongly expressed (Figure S2 related to Figure 1).^17^

To determine whether *Pax6os1*/*PAX6-AS1* expression may be modulated under conditions of glucotoxicity, levels of the lncRNA were measured in both murine and human cell lines as well as in primary islets maintained at different glucose concentrations. Culture for 48 h in the presence of high glucose induced *Pax6os1* expression in both MIN6 cells (15 and 35 vs. 5 mM glucose; *n* = 5, *p* = 0.02 and 0.03, respectively) and CD1 mouse islets (11 vs. 3 mM glucose; *n* = 3, *p* < 0.01) (Figures 1D and 1E). Furthermore, *Pax6os1* expression was increased in pancreatic islets from mice fed a high-fat diet (HFD) compared to controls (*n* = 6–5, *p* = 0.003), whereas *Pax6* mRNA levels remained unaffected (Figure 1F). Likewise, *PAX6-AS1* expression was upregulated in the human EndoC-βH1 cell line (*n* = 5, *p* = 0.01) as well as human pancreatic islets (*n* = 7, *p* = 0.03) cultured at elevated glucose concentrations (Figures 1G and 1H) (Table S1 related to Figures 1 and 6). More importantly, expression of *PAX6-AS1* was substantially (4- to 5-fold) increased in islets from donors with T2D (Hba1c ≥ 6.5% or fasting glucose ≥126 mg/dL) vs. normoglycemic donors (*n* = 11–5, *p* < 0.01) (Figure 1I). In contrast, *PAX6* mRNA levels remained constant independently of the glucose concentration or disease status (Figures 1G–1I).

### *Pax6os1* silencing upregulates beta cell signature genes in MIN6 cells

**Figure 2:**
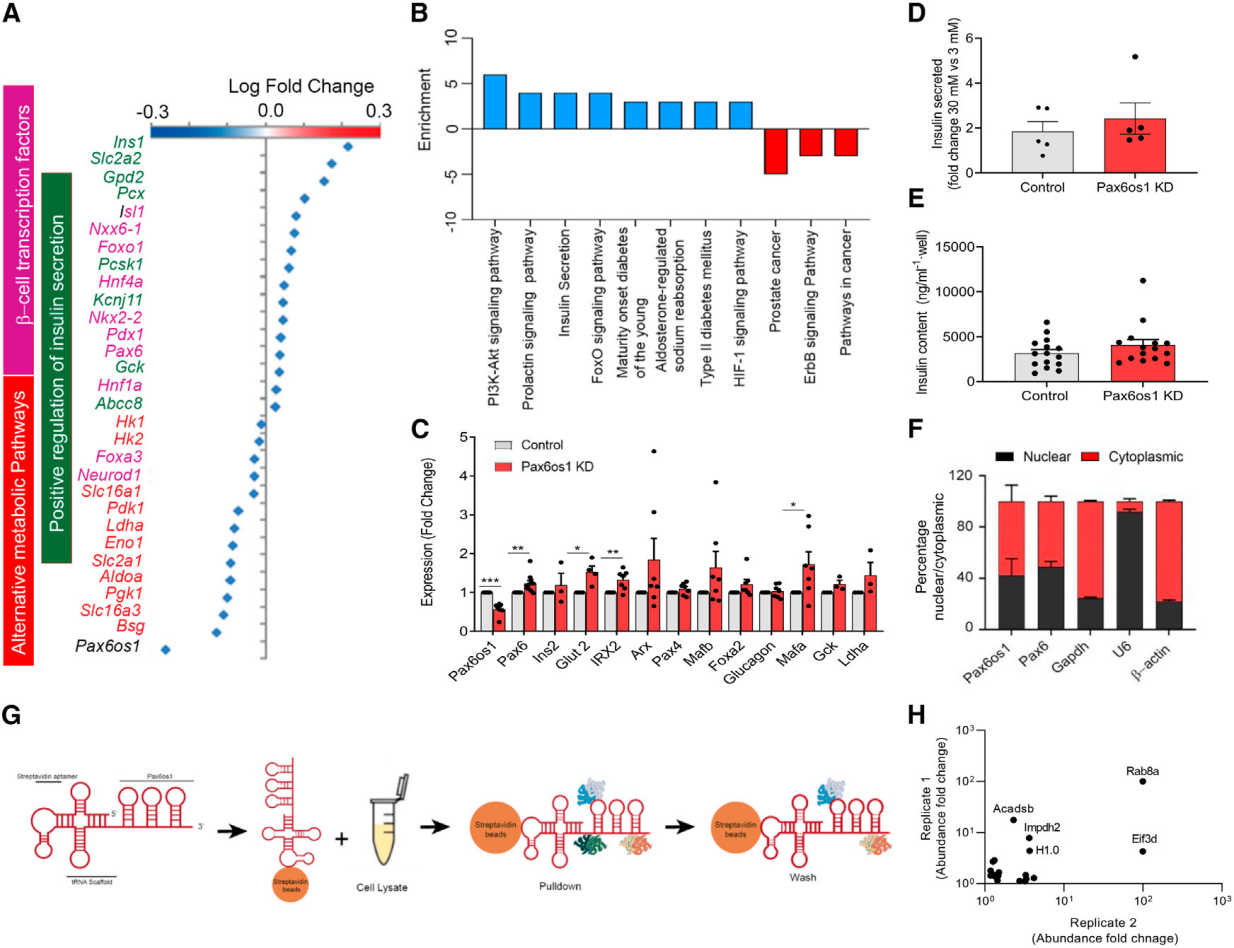
*Pax6os1* silencing upregulates beta cell signature genes in MIN6 cells (A) Differential expressed genes by *Pax6os1* knockdown as determined by RNA-seq performed in MIN6 cells 72 h post-transfection with siRNA targeting *Pax6os1*. *n* = 4. (B) KEGG pathway enrichment analysis relative to (A). Significantly enriched KEGG pathways (*p* < 0.05) are presented, and the bar shows the fold enrichment of the pathway. (C) mRNA levels of beta cell signature genes and markers characteristic of other endocrine cell lineages in control and *Pax6os1* knockdown cells. *n* = 7. (D) Fold change of insulin secreted relative to 3 mM glucose. *n* = 5. (E) Total insulin content. *n* = 14. (F) Pax6os1 subcellular distribution. (G) Schematic representation of Pax6os1 pull-down and MS. (H) Relationship in abundance ratios above the 1.1 cut between the two experimental replicates performed. Top 5 hits are labeled. Short/branched chain-specific acyl-CoA dehydrogenase, mitochondrial 1 (ACADSB), eukaryotic translation initiation factor 3 subunit D (EIF3D), inosine-5′-monophosphate dehydrogenase 2 (IMPDH2), histone 1.0 (H1.0), and uncharacterized protein Rab8a (Rab8a). Data are represented as the mean ± SEM. ∗*p* < 0.05, Student’s t test.

To explore the potential roles of *Pax6os1* in beta cell function or survival, we first transfected murine MIN6 cells with a small interfering RNA (siRNA) targeting the lncRNA. RNA sequencing (RNAseq) analysis was then performed, revealing that *Pax6os1* silencing (“knockdown”; KD) in MIN6 cells upregulated the expression of several beta cell signature genes, including *Ins1*, *Slc2a2 (Glut2)*, *and Pax6* while further downregulating several “disallowed genes” such as *Slc16a1* and *Ldha* (Figure 2A). In addition, enriched KEGG pathways in *Pax6os1*-silenced MIN6 cells included “insulin secretion,” “maturity onset diabetes of the young,” and “type II diabetes mellitus” (Figure 2B). RT-qPCR analyses in cells confirmed a 35 ± 5% decrease in *Pax6os1* expression (*p* = 0.0005) as well as an increase in *Pax6* (1.28 ± 0.046-fold change; *p* = 0.05), *Glut2*/*Slc2a2* (1.52 ± 0.15-fold change; *p* = 0.0144), and *Mafa* (1.72 ± 0.31-fold change; *p* = 0.040) mRNA levels (Figure 2C). However, despite the upregulation of several beta cell signature genes, glucose-stimulated insulin secretion (GSIS) was not affected by *Pax6os1* silencing (Figures 2D and 2E).

We next sought to determine the mechanism by which *Pax6os1* may modulate the expression of target genes in MIN6 cells. LncRNAs may regulate gene expression through a number of different mechanisms, including chromatin remodeling, activation/repression of transcription factors in the nucleus as well as modulation of mRNA/protein stability in the cytoplasm.^16^ Therefore, the subcellular localization of an lncRNA may provide a guide as to its mechanism(s) of action. Determinations of *Pax6os1* subcellular localization in MIN6 cells by subcellular fractionation indicated that the lncRNA was located in both the nucleus (∼40%) as well as the cytoplasm (∼60%) (Figure 2F). Consistent with these results, both nuclear and cytoplasmic proteins were identified by mass spectrometry as binding protein partners of *Pax6os1* (Table 1). Interestingly, the top five hits included Ras-related protein (RAB8A), eukaryotic translation initiation factor 3 subunit D (EIF3D), inosine-5′-monophosphate dehydrogenase 2 (Impdh2), short/branched chain-specific acyl-CoA dehydrogenase (ACADSB), and Histone H1.0 (Figures 2G and 2H). In addition, histones H4, H3.2, H2B, H1.1, and H1.4 in the nucleus as well as 3′-5′ RNA helicase YTHDC2 in the cytoplasm were also identified as *Pax6os1*-binding partners (Table 1).

**Table 1:** Pax6os1 protein binding partners identified by mass spectrometry

Name	% Protein coverage	Unique peptides	Average ratio (Control vs. Pax6os1)
Uncharacterized protein Rab8a	16.35	1	100
Eukaryotic translation initiation factor 3 subunit D	1.64	1	52.13
Histone H4	51.46	6	2.73
Histone H1.0	23.71	4	4.03
Inosine-5′-monophosphate dehydrogenase	3.89	2	5.71
Regulator of G-protein signaling 1	3.40	1	2.29
Histone H3.2	18.23	4	2.38
Histone H2B	27.40	5	2.18
Core histone macro-H2A.1	25.80	8	1.94
Short-/branched-chain-specific acyl-CoA dehydrogenase, mitochondrial 1	4.95	2	9.93
Histone H1.1	32.86	7	1.45
Pyrroline-5-carboxylate	3.43	1	1.30
Uncharacterized protein (fragment) Rpn2	2.12	1	1.36
DEAD (Asp-Glu-Ala-Asp) box polypeptide 23	5.25	4	2.07
3′-5′ RNA helicase YTHDC2	0.62	1	1.39
Spectrin beta chain, non-erythrocytic 1	0.76	2	1.98
Histone H1.4	50.68	5	1.31
Trifunctional enzyme subunit beta, mitochondrial	8.42	4	1.48

### *Pax6os1* deletion does not impact glucose homeostasis in T2D mouse models

**Figure 3:**
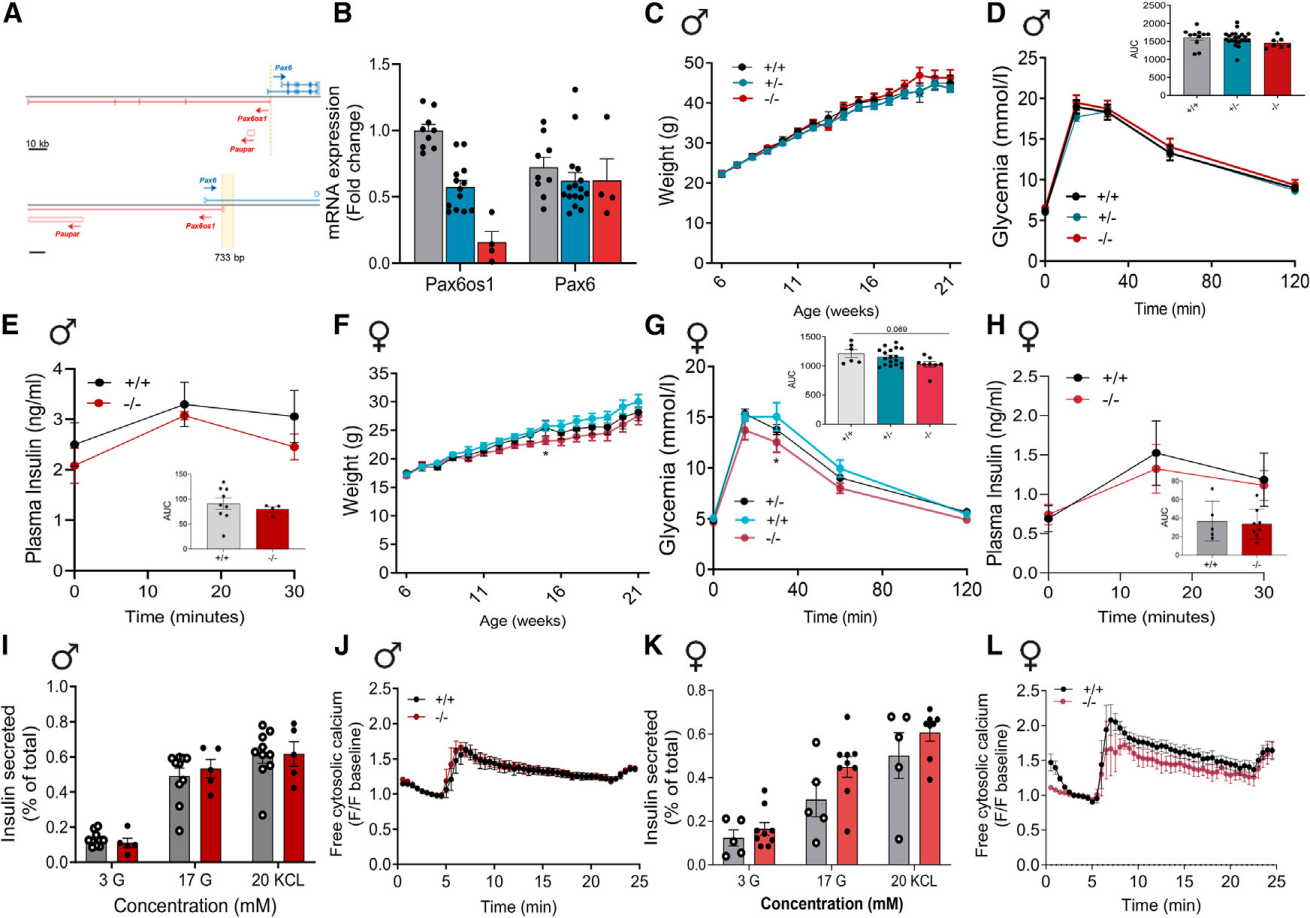
Pax6os1female null mice display mildly improved glucose tolerance and normal insulin secretion compared to WT animals under HFD (A) Schematic representation of the mutation generated in the Pax6os1 locus through CRISPR gene editing. (B) *Pax6os1* and Pax6 expression in islets isolated from wt (+/+), *Pax6os1* heterozygous (+/−), and *Pax6os1* homozygous (−/−) mice. (C) Body weights (g) of male Pax6os1 null mice under HFD. (D and E) Circulating glucose levels and insulin in plasma after receiving an intraperitoneal load of glucose in male Pax6os1 null mice. Glucose: wt (*n* = 11), *Pax6os1* +/− (*n* = 22), *Pax6os1* −/− (*n* = 6); insulin: wt (*n* = 9), *Pax6os1* (*n* = 4). (F) As (C) but in female mice. (G and H) As (D) and (E), respectively, but in female *Pax6os1* null mice. Glucose: wt (*n* = 6), Pax6os1 +/− (*n* = 18), Pax6os1−/− (*n* = 8); insulin wt (*n* = 6), Pax6os1 −/− (*n* = 8). (I) Insulin secreted (represented as % of the total) at different glucose concentrations and after depolarization with KCl in pancreatic islets isolated from male Pax6os1 null mice. *n* = 10–5. (J) Intracellular calcium in pancreatic islets isolated from male Pax6os1 null mice. *n* = 3.H. (K and L) As (I) and (J) for female mice. n = 5–9 (I); *n* = 3 (J). Data are represented as the mean ± SEM. ∗*p* < 0.05; two-way ANOVA repeated measurements.

In order to explore the possible consequences of *Pax6os1* loss for insulin secretion and glucose homeostasis *in vivo*, we used CRISPR/Cas9 gene editing to delete exon 1 of *Pax6os1* plus the immediate 5′ flanking region from the mouse genome in C57BL/6 mice (Figure 3A). Analysis of super-low input carrier-cap analysis of gene expression (SLIC-CAGE) data (unpublished) in mouse islets identified independent transcription start sites (TSS) for *Pax6* and *Pax6os1*, located ∼1 kb apart (Figure S3 related to Figure 3). Thus, the deletion generated spanned only the *Pax6os1* TSS and its putative promoter, as suggested by the presence of accessible chromatin in this region (ATAC-seq data) and of H3K4me3 ^24^ and H3K27Ac^24^ chromatin marks (Figure S3 related to Figure 3). Although *Pax6os1* expression was lowered by > 95% in islets from knockout (KO) mice, *Pax6* mRNA levels were unaffected (Figure 3B).

No statistically significant differences were observed *in vivo* between wild-type (WT) and *Pax6os1* KO male mice in weight, glucose clearance, insulin secretion under standard (STD) (Figure S4 related to Figure 3), or HFD (Figures 3C–3E). Similarly, no significant differences were observed between wild-type or *Pax6os1* KO female mice in STD diet in weight, glucose clearance, or insulin plasma levels (Figure S4 related to Figure 3). In contrast, *Pax6os1* KO female (but not male) mice under HFD displayed a tendency toward reduced body weight (Figure 3F) and significantly lower circulating glucose at 30 min (*p* = 0.041) during the IPGTT, with a strong trend toward a lower AUC during the experiment (WT: 1212 ± 169 a.u. vs. *Pax6os1* KO: 1030 ± 134 a.u. *p* = 0.069) (Figure 3G). However, no differences were observed in insulin secretion *in vivo* (Figure 3H). Furthermore, there were no significant differences in GSIS or intracellular calcium dynamics between islets isolated from *Pax6os1* KO mice and WT independently of sex in STD (Figure S4 related to Figure 3) or HFD (Figure 3I–3L).

In order to explore further the tendency observed in female mice in another animal model of T2D, we treated *db/db* male and female mice with antisense oligonucleotides (ASOs) targeting *Pax6os1*.^25^ A significant and near-significant downregulation of *Pax6os1* in pancreatic islets could be observed after 4 weeks of treatment with ASOs in female and male mice, respectively (Figure S5 related to Figure 3). However, no significant differences were observed in body weight or glucose clearance between the different experimental groups (Figures S5C–S5H).

### *PAX6-AS1* depletion in EndoC-βH1 cells induces the expression of beta cell signature genes

**Figure 4:**
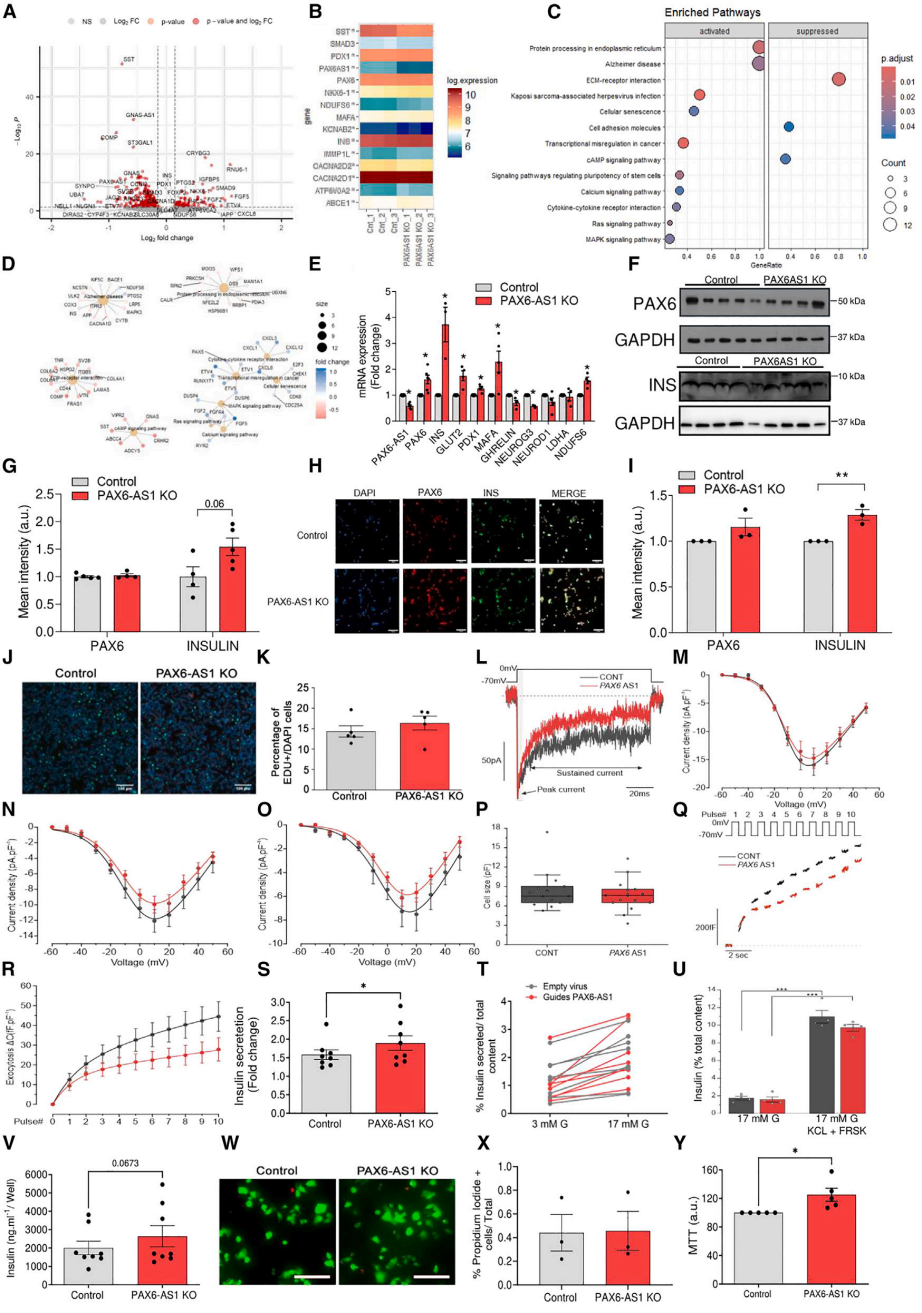
CRISPR/Cas9-mediated *PAX6-AS1* deletion in EndoC-βH1 cells increases insulin expression and enhances GSIS (A) Volcano plot representing genes significantly modulated in PAX6-AS1 depleted vs. control EndoC-βH1 cells. (B) Heatmap representing the log expression for selected genes. (C and D) Dotplot and cnetplot depicting the KEGG pathways significantly modulated in PAX6-AS1 KO EndoC-βH1 cells. (E) mRNA expression of *PAX6-AS1*, beta cell signature genes, and markers from other endocrine cell lineages in *PAX6-AS1*-deleted EndoC-βH1 cells. *PAX6-AS1, n* = 6; *PAX6, n* = 6; *INS*, *n* = 4; *GLUT2/SLC2A2*, *n* = 5; *PDX1*, *n* = 4; GHRL, *n* = 4; NEUROG3, *n* = 3; NEUROD1, *n* = 5; LDHA, *n* = 4. (F) Western blot showing Pax6 and insulin protein levels. (G) Densitometric analysis for (F). (H) Representative immunofluorescence images of control and PAX6-AS1 KO EndoC-BH1 cells stained for PAX6 and insulin. Scale bar: 100 μm. (I) Mean intensity for PAX6 and insulin staining. (J and K) Proliferation in PAX6-AS1-deleted EndoC-βH1 cells assessed by EdU staining: representative images (J) and quantification (K, *n* = 5). At least 1,000 cells were counted per experiment using ImageJ software. Scale bar: 100 μm. (L) Raw traces of mixed sodium and calcium currents elicited by a 100 ms depolarization from −70 to 0 mV in PAX6-AS1 KO and control cells. (M–O) Quantification of the current density (see STAR Methods) at the peak, which is mainly composed of the rapidly inactivating voltage-gated sodium and calcium currents (M), at 5 ms when only the calcium component remains (the sodium component is inactivated) (N), and for the sustained component (O). *n* = 15. (P) Cell size for control and PAX6-AS1 KO EndoC-βH1 cells. *n* = 15 cells in both cell types. (Q) Cumulative exocytosis was determined upon 10 depolarizations (pulses) from −70 to 0 mV (top panel) using membrane capacitance measurement. (R) Quantification of the cumulative exocytosis at each pulse. *n* = 14–13. (S and T) Fold change of glucose-induced insulin secretion (S) and insulin secreted as percentage of total content (T). *n* = 8. (U) Insulin secretion induced by 17 mM glucose and with addition of 35 mM KCl and 25 μM forskolin (FRSK). (V) Determination of total insulin content per well. *n* = 8. (W and X) Representative images showing calcein (green) and propidium iodide (red) staining and quantification of the percentage of propidium-iodide-positive cells. *n* = 3. At least 1,000 cells were counted per experiment using ImageJ software. Scale bar, 100 μm. (Y) MTT assay. *n* = 5. Data are represented as the mean ± SEM. ∗*p* < 0.05; Student’s t test or two-way ANOVA.

In order to determine the effect of *PAX6-AS1* KO in human beta cells, we used a tailored CRISPR/Cas9 approach to delete ∼80 bp within the first exon of *PAX6-AS1* from fetal-human-pancreas-derived EndoC-βH1 cells (Figure S6 related to Figure 4). Despite previously mentioned differences between *PAX6-AS1* and its ortholog in mouse, an RNA-seq analysis revealed several genes commonly modulated after silencing the lncRNA in MIN6 and EndoC-βH1 cells. Indeed, *PAX6-AS1*-depleted EndoC-βH1 cells displayed increased expression of the beta cell signature genes, *INS*, *PDX1*, and *NKX6-1* (Figures 4A and 4B). However, no differences were observed in the expression of *LDHA* and *SLC16A1*, although the disallowed gene *SMAD3* was downregulated in *PAX6-AS1* KO compared to control cells. Further supporting the hypothesis that *PAX6-AS1* depletion favors the expression of beta cell signature genes over genes typically expressed in other endocrine cell types, somatostatin (SST) expression was robustly reduced in *PAX6-AS1* KO cells (Figures 4A and 4B). Interestingly, although the calcium channels *CACNA2D1* and *CACNA2D2* were downregulated in *PAX6-AS1*-depleted cells (Figures 4A and 4B), calcium signaling appeared as one of the KEGG pathways significantly activated after *PAX6-AS1* depletion due to the upregulation of RYR2 and FGF5 (Figures 4C and 4D). Other activated pathways included protein processing in the endoplasmic reticulum and the mitogenic secondary branch of insulin signaling Ras-MAPK (Figures 4C and 4D), whereas cAMP signaling and ECM-receptor interaction pathways were suppressed (Figures 4C and 4D). Increased expression of *INS* (2.727 ± 0.6649-fold change, *p* = 0.01), *PDX1* (0.2625 ± 0.07348-fold change, *p* = 0.01), and NDUFS6 (0.5690 ± 0.1227-fold change, *p* = 0.003) was confirmed by RT-qPCR in PAX6-AS1-depleted cells (Figure 4E). Intriguingly, several genes that did not appear to be significantly modulated by *PAX6-AS1* depletion in our RNA-seq data, such as PAX6 (*p* = 0.009; p-adj = 0.15), showed increased mRNA levels in *PAX6-AS1* KO compared to control cells when measured by RT-qPCR (1.60 ± 0.16-fold change, *p* = 0.002). However, no differences in *PAX6* protein levels were observed as measured by western blot or immunofluorescence (Figures 4F–4I), indicating that *PAX6* changes are unlikely to underlie the phenotype observed after *PAX6-AS1 deletion*. In contrast, *PAX6-AS1* KO cells displayed a strong tendency toward higher protein insulin levels when measured by western blot (0.5438 ± 0.23-fold change, *p* = 0.057) (Figures 4F and 4G), which was significant when measured by immunofluorescence (Figures 4H and 4I).

In spite of the activation of the mitogenic Ras-MAPK pathway, no differences were observed in the proliferation between control and *PAX6-AS1* KO EndoC-βH1 cells as determined by EdU staining (Figures 4J and 4K). Similarly, no significant differences were found in calcium currents between different cell types, which displayed similar cell size (Figures 4L–4P). A strong tendency toward reduced exocytosis consistent with cAMP pathway suppression could also be observed in *PAX6-AS1* KO cells (Figures 4Q and 4R). In contrast, *PAX6-AS1* depletion slightly enhanced GSIS in EndoC-βH1 cells as determined by an increased fold change in insulin secretion between 0.5 mM and 17 mM glucose (Control: 1.584 ± 0.13, PAX6-AS1 KO: 1.894 ± 0.19; *p* = 0.041) (Figures 4S and 4T) but not when cells were directly depolarized with KCl (Figure 4U).

Remarkably, the expression of SVB2 (Figures 4A and 4D), which is involved specifically in the exocytosis of GABA-containing synaptic-like microvesicles but not in insulin release,^26^^,^^27^ was reduced in *PAX6-AS1* KO cells, whereas other genes involved in acidification and vesicular trafficking such as *ATPV0A2*^28^^,^^29^ were upregulated (Figures 4A and 4B). The improvement in GSIS was also accompanied by a strong tendency toward increased insulin content (Figure 4V) with no variations in cell number as suggested by the lack of significant differences in proliferation (Figures 4J and 4K) or cell death (Figures 4W and 4X). Furthermore, *PAX6-AS1*-depleted cells displayed increased mitochondrial activity as indicated by MTT assay (Figure 4Y) and the upregulation of *NDUFS6* and *IMMP1L* (Figures 4B and 4E).

### *PAX6-AS1* directly interacts with histones and EIF3D

**Figure 5:**
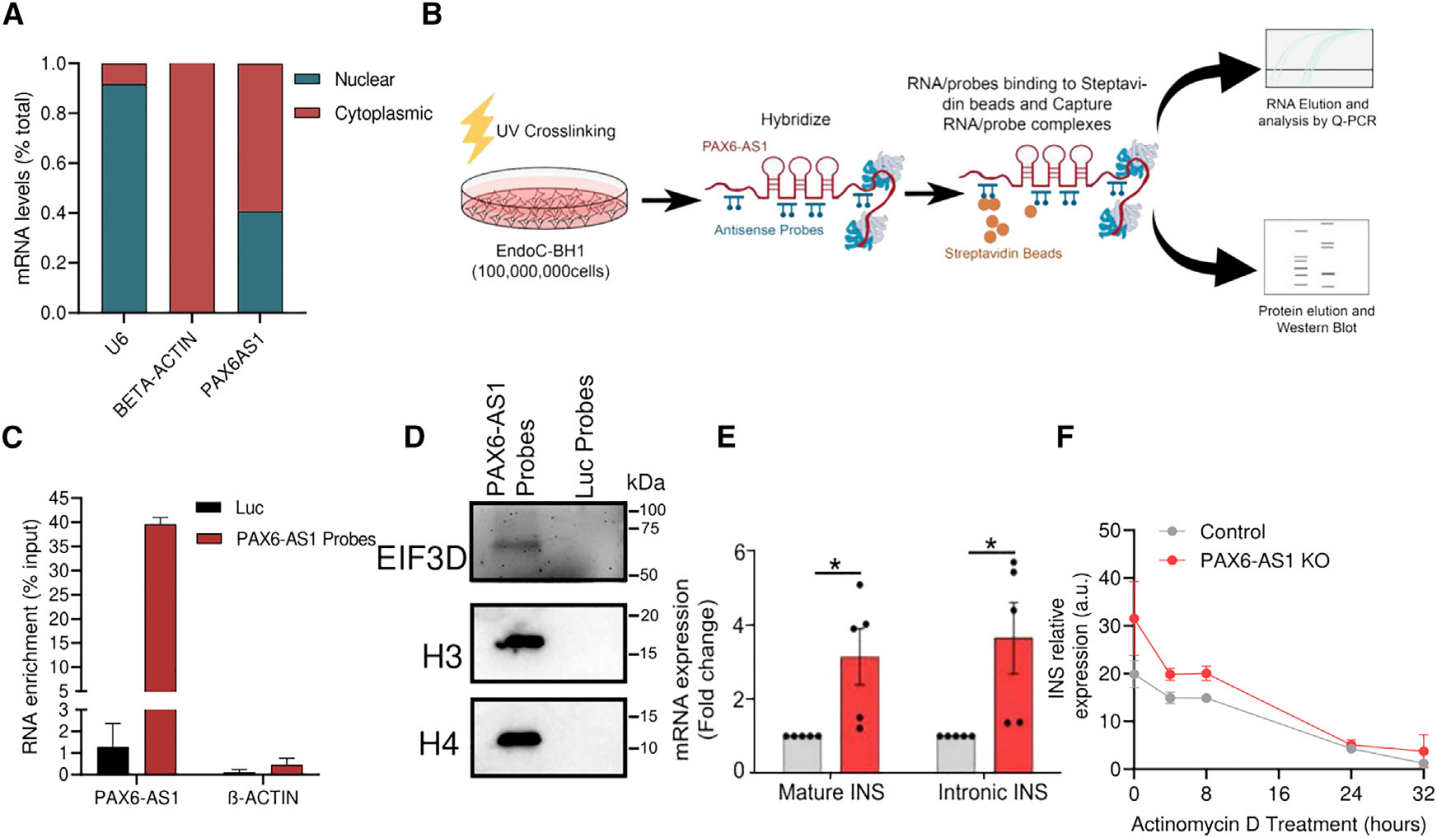
*PAX6-AS1* directly interacts to EIF3D, H3, and H4 (A) Subcellular localization of PAX6-AS1 in EndoC-βH1 cells. *n* = 3. (B) Schematic representation of the RNA antisense pull-down. (C) PAX-AS1 mRNA enrichment vs. input in cells hybridized with probes targeting the lncRNA. (D) Western blots showing EIF3D, H3, and H4 in cells hybridized with probes against our lncRNA or luciferase. (E) Mature and nascent insulin mRNA expression as determined by qPCR in control and PAX6-AS1 depleted cells. *n* = 5. (F) Determination of insulin mRNA stability as determined by actinomycin D treatment in control and PAX6-AS1 KO cells. n = 6–3. Data are represented as the mean ± SEM. ∗*p* < 0.05, Student’s t test.

In order to identify the molecular mechanisms of action of PAX6-AS1, we next sought to determine its subcellular localization in EndoC-βH1 cells. Remarkably, PAX6-AS1 displayed the same expression pattern than Pax6os1, being located in the nucleus and the cytoplasm at similar proportions (∼40% and ∼60%, respectively) (Figure 5A). Next, we sought to validate in the human cell line the binding partners previously identified by mass spectrometry in MIN6 cells. To this end, we performed an RNA antisense pull-down (RAP), using biotinylated DNA probes antisense to our lncRNA followed by western blot analysis (Figure 5B).^30^ Successful RNA pull-down was confirmed by RT-qPCR in the RNA elution fraction, obtaining a ∼40% PAX6-AS1 enrichment vs. input using four specific probes targeting our lncRNA (Table S4 related to Figure 5) (Figure 5C). In contrast, only 1.5% PAX6-AS1 enrichment vs. input was observed in the control group hybridized with probes targeting luciferase. Both groups showed <0.002% β-ACTIN enrichment vs. input, confirming the specificity of our probes (Figure 5C). A direct interaction between PAX6-AS1 and EIF3D, H3, as well as H4 was confirmed by western blot (Figure 5D), whereas direct binding of the lncRNA to other partners previously identified such as H1 could not be confirmed (data not shown). These results suggest that PAX6-AS1 may regulate protein translation^31^ as well as transcription of target genes. In line with these results, PAX6-AS1 seemed to regulate INS expression at the transcriptional level as determined by increased levels of INS nascent mRNA and the lack of significant differences in mRNA stability between control (INS half-life: 11.29 ± 4.21 h) and PAX6-AS1-depleted cells (INS half-life: 9.04 ± 1.7 h) (*p* = 0.46) after treatment with actinomycin D (5 μg/mL) (Figures 5E and 5F).

### *PAX6-AS1* knockdown enhances, whereas overexpression impairs, GSIS from human islets

**Figure 6:**
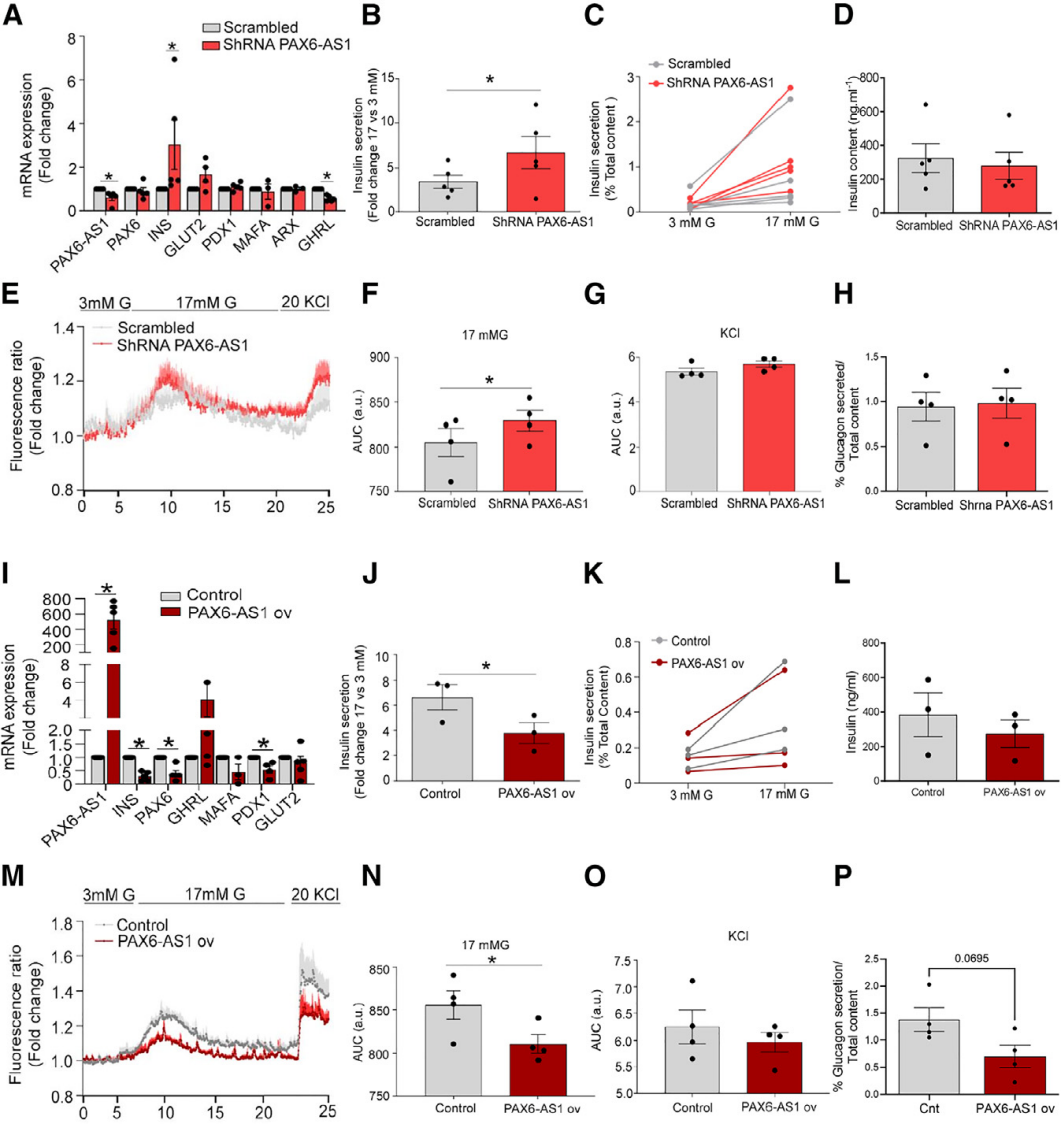
*PAX6-AS1* knockdown in human islets enhances GSIS, whereas *PAX6-AS1* overexpression exerts opposite effects (A) mRNA expression of *PAX6-AS1*, beta-signature genes, and markers from other endocrine cell lineages in islets infected with a scrambled or a shRNA-targeting PAX6-AS1. n = 4–5. (B and C) GSIS represented as the fold change or % of total insulin content in *PAX6-AS1*-silenced islets. *n* = 5. (D) Total islet insulin content. (E) Trace showing calcium response in *PAX6-AS1*-silenced islets. *n* = 4. (F and G) Area under the curve (AUC) for calcium dynamics in response to 17 mM glucose and 20 mM KCl, respectively, in *PAX6-AS1*-silenced islets. *n* = 4. (H) Glucagon secretion in *PAX6-AS1*-silenced islets compared to scrambled. *n* = 4. (I) mRNA expression of PAX6-AS1 and beta cell signature genes in control and islets overexpressing PAX6-AS1. n = 6–3. (J and K) GSIS represented as the fold change or % of total insulin content in *PAX6-AS1*-overexpressing islets. *n* = 3. (L) Total islet insulin content. (M) Trace showing calcium response in *PAX6-AS1*-overexpressing islets. *n* = 4. (N and O) Area under the curve (AUC) for calcium dynamics in response to 17 mM glucose and 20 mM KCl, respectively, in *PAX6-AS1*-overexpressing islets. *n* = 4. (P) Glucagon secretion in PAX6-AS1-overexpressing islets compared to control. *n* = 4. Data are represented as mean ± SEM. ∗*p* < 0.05, paired Student’s t test.

To extend our results to fully differentiated human beta cells, we used lentiviral shRNA vectors to silence *PAX6-AS1* in pancreatic islets from postmortem donors (Table S1, related to Figures 1 and 6). Transduced islets displayed a reduction in *PAX6-AS1* expression of 49 ± 12%, increased *INS* mRNA levels (2.727 ± 0.6649-fold change, *n* = 5, *p* = 0.04), and reduced *GHRL* expression (0.57 ± 0.05-fold change, *p* < 0.0001) (Figure 6A). Importantly, the upregulation in INS mRNA levels was accompanied by enhanced GSIS (Scrambled: 3.44 ± 0.74-fold change vs. *PAX6-AS1* shRNA: 6.69 ± 1.78-fold change, *n* = 5, *p* = 0.03), although total insulin content was not affected (Figures 6B–6D). *PAX6-AS1*-silenced islets also showed increased intracellular Ca^2+^ dynamics in response to 17 mM glucose as assessed by the AUC for mean fluorescence (scrambled: 13.09 ± 0.16 a.u. vs. *PAX6-AS1* shRNA: 13.69 ± 0.10 a.u., *p* = 0.049, paired t test) (Figures 6E and 6F). In contrast, Ca^2+^ responses to plasma membrane depolarization with KCl, added to open voltage-gated Ca^2+^ channels directly, were not significantly affected by *PAX6-AS1* silencing (Figure 6G). No additional effect was observed on glucagon secretion elicited by 1 mM glucose, suggesting that *PAX6-AS1* downregulation does not affect alpha cells (Figure 6H).

Demonstrating a deleterious effect on human beta cell function, *PAX6-AS1* overexpression in human islets led to a strong reduction in *INS* (0.29 ± 0.08-fold change, *p* < 0.0001) and *PDX1* (0.54 ± 0.14-fold change, *p* = 0.008) expression, whereas *GHR* was not affected (Figure 6I). The decrease in the expression of beta cell signature genes was accompanied by impaired GSIS (Control: 3.32 ± 0.5-fold change vs. *PAX6-AS1* overexpression: 1.89 ± 0.41-fold change, *p* = 0.02) but unaltered total insulin content (Figures 6J–6L). Correspondingly, *PAX6-AS1*-overexpressing islets displayed a significant reduction in intracellular Ca^2+^ dynamics in response to 17 mM glucose (Control: 16.05 ± 1.03 a.u. PAX6-AS1 overexpression: 15.01 ± 1.085, *p* = 0.01, paired t test) (Figures 6M and 6N), whereas there were no significant differences in the response to depolarization with KCl (Figure 6O). In line with silencing experiments, overexpressing *PAX6-AS1* in islets did not significantly affect alpha cell functionality. Nevertheless, a tendency toward decreased glucagon secretion could be observed, suggesting that although this lncRNA is not normally expressed in alpha cells, forcing its expression can be detrimental in all pancreatic endocrine cells (Figure 6P).

## Discussion

We show that *Pax6os1*/*PAX6-AS1*, a lncRNA transcribed from the *Pax6* locus and previously identified in the murine retina,^18^ is chiefly expressed in beta cells within the pancreatic islet. This distribution differs from that of *Paupar*, also expressed from the *Pax6* locus, which is largely confined to alpha cells.^17^ We demonstrate that *Pax6os1* is upregulated at high glucose concentrations, in an animal model of T2D (HFD) and in pancreatic islets from patients with this disease. Thus, it is tempting to speculate that increased expression of *PAX6-AS1* contributes to the pathogenesis of T2D. Further detailed studies will be needed to determine how, at the molecular level, glucose or other factors that contribute to this disease, such as fatty acids, affect *Pax6os1*/PAX6AS1 expression.

Supporting this hypothesis, *Pax6os1* silencing increased the expression of several beta cell signature genes in murine MIN6 cells, while decreasing mRNA levels of disallowed genes, suggesting a role for the lncRNA in beta cell identity. However, no significant differences were found in GSIS *in vitro.* Additional experiments, including an exploration of chromatin accessibility and transcription factor binding to relevant genomic sites, ultrastructural, proteomic, or other studies may nevertheless be useful in the future to more fully explore the impact of *Pax6os1* on beta cell identity. Mice in which *Pax6os1* was deleted *in utero* displayed only a modest phenotype, which was only evident in females maintained on an HFD diet.

Dissecting the functions of different transcripts within complex *loci* such as *Pax6/Pax6os1* is inherently challenging due to the close proximity of the transcriptional start sites. In this regard, it was conceivable that the DNA fragment deleted from the mouse genome (720 bp) by CRISPR/Cas9 to generate the *Pax6os1* KO mouse might interfere with a regulatory region of *Pax6*, directly affecting the expression of the transcription factor. Indeed, assessment of the open chromatin state (by ATACSeq), and regulatory histone marks, indicated that the deletion of *Pax6os1* exon 1 and the proximal promoter region might potentially exert an effect on *Pax6* expression *in cis*. Furthermore, it was also possible that early developmental compensation occurred *in vivo* after *Pax6os1* deletion in the mouse, minimizing the effects on beta cell function. However, db/db mice with *Pax6os1* silenced by antisense oligonucleotides at 6 weeks of age showed no phenotype in glycemic control, arguing against an effect mediated by direct alterations in Pax6 levels by our CRISPR technique or an early developmental compensation. Furthermore, the effects of *Pax6os1* inactivation were only observed in female mice, whereas Pax6 changes are supposed to affect both sexes equally. The absence of phenotype in db/db female mice might be explained due to the fact that treatment was started when mice were already hyperglycemic at fasting.

In line with our results in MIN6 cells and in female mice, deletion of *PAX6-AS1* in the human beta cell line, EndoC-βH1, upregulated the expression of several beta cell signature genes. Nevertheless, important differences were found between mouse and human cell lines. For instance, insulin was stronger regulated by PAX6AS1 in humans, which could partially explain the different phenotype observed between the two species in GSIS after deletion of the lncRNA. Actually, protein insulin levels were only altered in the human cell line. Therefore, the lack of differences in insulin protein levels could underlie, at least to some extent, the absence of a major phenotype in mice *in vivo*. In addition, PAX6 in humans was not robustly modulated, finding only significant differences in the RT-PCR but not in the RNA-seq or protein levels. These discrepancies between diverse methods could be partially due to differences in sample size and statistical power. However, it could also indicate that PAX6 and other genes may be only slightly modulated by PAX6AS1 depending on the cell-cycle state. Despite tendencies toward decreased calcium currents and decreased exocytosis, GSIS was slightly enhanced by *PAX6-AS1* deletion in EndoC-βH1 cells. This improvement in insulin release was accompanied by an apparent increase in mitochondrial activity and gene expression, which was not observed in mice, representing another important difference between species that could affect GSIS. Enhanced GSIS was also observed after lentivirus-mediated *PAX6-AS1* knockdown in human islets, and this was accompanied by increased calcium dynamics in response to glucose. In contrast, islets overexpressing the lncRNA displayed impaired GSIS and cytosolic calcium dynamics. We note that *PAX6-AS1* is expressed in delta cells, albeit at lower levels than in beta cells, and that SST expression was strongly reduced in EndoC-βH1 cells. Therefore, impaired somatostatin secretion from delta cells after *PAX6-AS1* knockdown in islets may contribute to the direct effects of inactivating the lncRNA in the beta cell, further enhancing GSIS.

RNA pull-down experiments revealed that, among other partners, *Pax6os1/PAX6-AS1* binds the histones H3 and H4. Furthermore, Pax6os1/PAX6AS1 was shown to also bind EIF3D, which is a driver of noncanonical-cap-dependent translation of specific mRNAs. These results suggest that Pax6os1/PAX6AS1 regulates the expression of target genes by modulating transcription through epigenetic modulations as well as translation of specific mRNAs. Interestingly, in humans, EIF3D has been shown to be especially important for the translation of proteins involved in metabolism and metabolic stress caused by glucose deprivation.^31^ Therefore, it is tempting to speculate that some of the metabolic alterations observed in human cells and islets after PAX6AS1 silencing or overexpression are mediated by the interaction between PAX6AS1 and EIF3D.

Our data suggest that *Pax6os1/AS1* affects beta cell signature genes in both mice and humans and that they share several binding partners. Nevertheless, important species differences were also found, with more marked effects observed in humans than in mice. Interestingly, the effects of *Pax6os1* deletion were sex-dependent in mice. Whether such differences also pertain in humans could not readily be explored here given the relatively small number of islet samples available and the use a cell line (EndoC-βH1) from only one sex (female).^32^

LncRNAs have emerged in recent years as promising therapeutic targets in several diseases.^33^^,^^34^ We show here that PAX6-AS1 silencing in islets enhances insulin secretion, whereas increased expression of PAX6-AS1—as observed in T2D—may contribute to beta cell dysfunction and impaired GSIS. Although levels achieved in overexpression experiments were beyond the (patho-) physiological range, even in overexpressing cells, PAX6-AS1 mRNA levels were still relatively low compared to most mRNAs, and thus a non-specific toxic effect seems unlikely. Targeting PAX6-AS1 might therefore provide a novel approach to maintain beta cell functionality in T2D. Experiments that explore the impact of modulating *PAX6-AS1* expression in islets from T2D patients may provide useful insights into the possible clinical value of this approach.

### Limitations of the study

One of the limitations of this study is the high level of overexpression of PAX6-AS1 achieved in islets, which do not represent physiological levels of the lncRNA. However, although some of the observed alterations in mRNA levels may be caused by the extraordinary high levels of PAX6-AS1, it is important to note that other significant changes such as impairment in GSIS and reduced cytoskeletal calcium dynamics are also observed in the opposite direction, with only a 40% reduction in the expression of this lncRNA. Another important caveat of this study is the mild phenotype observed in mice, which points to important species differences, but strongly limits the possibilities of studying the role of this lncRNA in a physiological setting *in vivo*.

## Resources availability

### Lead contact

Further information and requests for resources should be directed to and will be fulfilled by the lead contact, Professor Guy A. Rutter (g.rutter@imperial.ac.uk).

### Materials availability

Pax6os1 KO mice are conserved as frozen embryos at Imperial College facilities and available upon request.

### Data and code availability

• RNA sequencing data have been deposited at ENA (MIN6) or SRA (EndoC-βH1) and are publicly available as of the date of publication. Proteomic data have been deposited at PRIDE. Data accession numbers are described in the key resources table.
• No original code is reported in this manuscript.
• All other data reported in this paper and any additional information required to reanalyze it will be shared by the lead contact upon request.

## Acknowledgments

G.A.R. was supported by Wellcome Trust Senior Investigator (WT098424AIA) and Investigator (WT212625/Z/18/Z) Awards, MRC Programme grant (MR/R022259/1), Diabetes UK (BDA/11/0004210, BDA/15/0005275, BDA 16/0005485), Imperial Confidence in Concept (ICiC) grants, an NIH-NIDDK project grant (R01DK135268), a CIHR-JDRF Team grant (CIHR-IRSC TDP-186358 and JDRF 4-SRA-2023-1182-S-N), CRCHUM start-up funds, and an Innovation Canada John R. Evans Leader Award (CFI 42649). G.A.R. and P.M. received funding from the European Union’s Horizon 2020 research and innovation programme via the Innovative Medicines Initiative 2 Joint Undertaking under grant agreement No 115881 (RHAPSODY). A.M.-S. was supported by an MRC New Investigator Research Grant (MR/P023223/1). B.H. was supported by RD Lawrence fellowship (BDA number:19/0005965). This project has received funding from the European Union’s Horizon 2020 research and innovation program via the Innovative Medicines Initiative 2 Joint Undertaking under grant agreement No 115881 (RHAPSODY) to G.A.R. and P.M. B.R.G. is supported by 10.13039/501100004837Ministerio de Ciencia e Innovación de España (PID2021-123083NB-I00 financed by MCIN/AEI/10.13039/501100011033 and by FEDER, UE) and the Juvenile Diabetes Research Foundation (3-SRA-2023-1307-S-B). L.L.N. is supported by the Consejería de Economía, Innovación y Ciencia (DOC_00652, FSE and Lema: Andalucía moves with Europe). The E.J.P. de K. lab is financially supported by the 10.13039/501100003092Dutch Diabetes Research Foundation, 10.13039/501100011570DON Foundation, Tjanka Foundation, 10.13039/501100005040Bontius Foundation, and RegMedXB. We thank the Oxford Biomedical Research Center (BRC), Diabetes Research and Wellness Foundation (DRWF), and Juvenile Diabetes Research Foundation (JDRF) for the provision of human islets. Human islets for research were also provided by the Alberta Diabetes Institute IsletCore at the University of Alberta in Edmonton (http://www.bcell.org/adi-isletcore.html) with the assistance of the Human Organ Procurement and Exchange (HOPE) program, Trillium Gift of Life Network (TGLN), and other Canadian organ procurement organizations. Islet isolation was approved by the Human Research Ethics Board at the University of Alberta (Pro00013094). All donors' families gave informed consent for the use of pancreatic tissue in research.

## Author contributions

L.L.N. and R.C. performed most of the experiments and analyzed data. A.M.-S. contributed to experiments with human islets. G.P. prepared RNAseq libraries. A.M.-S., N.H., N.C., and B.L. generated SLIC-CAGE and performed the analysis of ATAC-seq and histone marks in mouse islets. S.N. and B.H. performed electrophysiology. P.M., L.P., E.K., A.M.J.S., and P.J. provided human islets. M.-S.N. and I.L. assisted with *in vivo* work. L.L.N. and G.A.R. wrote the manuscript. B.R.G., R.C., and A.M.-S. critically reviewed the manuscript. T.J.P. and G.A.R. conceived and supervised the study.

## Declaration of interests

G.A.R. has received grant funding and consultancy fees from Sun Pharmaceuticals Inc. The remaining authors declare no conflict of interest.

## STAR★Methods

### Key resources table

**Table:** 

REAGENT or RESOURCE	SOURCE	IDENTIFIER
**Antibodies**		

Anti-Pax6	Biolegend	PRB-278P; RRID: AB_291612
Anti-GAPDH	Cell Signaling	2118; RRID: RRID: AB_561053
Anti-H4	Cell Signaling	2935; RRID: AB_1147658
Anti-H3	Sigma	H0164-25UL; RRID: AB_532248
Goat Anti-Rabbit (HRP)	Abcam	ab6721; RRID: AB_955447
Rabbit Anti-mouse (HRP)	Sigma	A9044; RRID: AB_258431
Anti-Insulin	Cell Signaling	8138; RRID: AB_10949314
Anti-EIF3D	Proteintech	10219-1-AP; RRID: AB_2096880

**Bacterial and virus strains**		

lentiCRIPSR v2	Sanjana, N.E. et al.^35^	Addgene #52961
Lentivirus ORF expression (CMV promoter)	Amsbio	NA (custom)
Lentivirus shRNA expression	Amsbio	NA (custom)

**Biological samples**		

Human pancreatic Islets	See Table S1	See Table S1

**Chemicals, peptides, and recombinant proteins**		

Cal-520	Abcam	ab171868
Calcein-AM	Molecular Probes	56496-20X50UG
Propidium iodide	Sigma-Aldrich	25535-16-4
Biotin	Sigma-Aldrich	B4501

**Critical commercial assays**		

Click-iT EdU Alexa Fluor 488 HCS Assay	ThermoFisher	C10350
Homogeneous Time Resolved Fluorescence (HTRF) insulin assay kit	Cisbio	62IN1PEG
Glucagon ELISA kit	Mercodia	10-1271-01
Mouse insulin ELISA	CrystalChem	90080

**Deposited data**		

MIN6 RNA-seq data	This paper	ENA: PRJEB38858
EndoC-BH1	This paper	SRA: PRJNA1105640
Mass spectrometry data of Pax6os1 binding partners	This paper	PRIDE: PXD052133

**Experimental models: Cell lines**		

MIN6 cells	Miyazaki, J. et al.^23^	NA
Endoc-βH1 cells	Tsonkova, V.G. et al.^35^	NA

**Experimental models: Organisms/strains**		

Pax6os1 null mice	This paper	NA
Db/db mice	Charles River	000697

**Oligonucleotides**		

Primers used in this study are described in Table S3.	Sigma	NA

**Recombinant DNA**		

pX330-U6-Chimeric_BB-CBh-hSpCas9 plasmid	Cong, L. et al.^24^	Addgene #42230
lentiCRIPR v2	Sabjana NE et al.^25^	Addgene #52961
psPAX2	Addgene	Addgene # 12260
pMD2.G	Addgene	Addgene #12259
Biotin labeled DNA probes (see Table S4)	IDT	NA (custom)

**Software and algorithms**		

STAR 2.7	Dobin, A. et al.^27^	NA
HTseq 2.0	Putri, G.H. et al.^28^	NA
DESeq2	Love, M.I. et al.^29^	NA
ClusterProfiler	Xu, S. et al.^30^	NA

**Other**		

Guide RNAS (see Table S2)	This paper	NA

### Experimental model and study participant details

#### Human islets

Islets were isolated from the pancreas of cadaveric human donors at the Isolation Centers stated in Table S1 related to Figures 1 and 6. Islets were used with the approval of the Human Research Ethics Board at the University of Alberta, the Oxfordshire Regional Ethics Committee B, the Ethics Committee from San Raffaele Hospital (Milan), or e Ethics Committee of the University of Pisa, upon written consent of donors’ next-of-kin. All families of organ donors provided written informed consent for use in research. Race and ethnicity were not provided, while age, sex and other relevant information such as BMI are described in Table S1 related to Figures 1 and 6. Islets were shipped in CMRL media (Thermo Fisher Scientific). Upon arrival islets were cultured in RPMI-1640 (11879-020) supplemented with 5.5 mM glucose, 10% FBS, 1% penicillin/streptomycin and 0.25 μg/mL fungizone. For each donor, islets were randomly distributed into different groups. Islets from donors 60, 74, 80, 85, 95, 106, 114 were used for different glucose concentration studies. For PAX6-AS1 expression studies in T2D, islets from donors 60, 74, 80, 85, 95, 106, 114, 116, 165,177 were used as control, while donors 49, 78, 91, 101, 127 were reported to have T2D. Islets from donors 166, 177, 178, 182 and 188 were randomly distributed into two groups and infected with Scrambled or PAX6-AS1 shRNA lentiviruses for GSIS and expression studies. Islets from donors 189, 190, 193 and 196 were divided randomly into two groups and infected with lentiviruses harboring an empty vector or PAX6-AS1 cDNA. Islets from donors R480, R474, R481 and R485 were randomly allocated into 4 groups and infected with lentiviruses harboring either PAX6-AS1 shRNA. PAX6-AS1 cDNA or their corresponding controls to perform glucagon secretion studies.

#### Animals

All animal procedures were performed with approval from the EU Directive 2010/63/EU for animal experiments. Pax6os1 null mice experiments were performed with the approval of the British Home Office under the UK Animal (Scientific Procedures) Act 1986 (Project License PPL PA03F7F07 to I.L.) and from the local ethical committee (Animal Welfare and Ethics Review Board, AWERB) at Imperial College London. Db/db mice procedures were approved by the Andalusian Regional Ministry of Agriculture, Fish, Water and Rural Development (#3/11/2021/171/A and ALURES #nts-es-414463) and performed in accordance with the Spanish law on animal use RD 53/2013. Animals were housed in individually ventilated cages and kept under controlled environmental conditions (12 hs-light–dark cycle, 23 ± 1°C with 30–50% relative humidity). Mice were provided with standard rodent chow, unless stated otherwise, and sterilized tap water *ad libitum*. For the HFD study, a chow enriched with 58% Fat and 25% Sucrose diet (D12331, Research Diet, New Brunswick, NJ) was used. Db/db mice were injected intraperitoneally either with scrambled or antisense oligonucleotides (5 mg/kg) purchased from QIAGEN.^25^ Mice were randomly allocated in two groups at the beginning of the treatment. All experiments were performed in male and female adult mice (6–12 weeks old). Animals of each sex were analyzed separately to determine the potential influence on phenotype. In all studies, littermates were used as controls.

#### Cell lines

MIN6 cells (established from an insulinoma developed in an IT6 transgenic C57BL/6 male mouse)^36^ were cultured with in DMEM (Sigma-Aldrich) (25mM glucose) supplemented with 15% (v/v) fetal bovine serum (FBS) and 2 mM L-glutamine.

EndoC-βH1 cells (derived from a female human fetus)^32^ were cultured in DMEM medium (Thermo Fisher, 31885023) (5.5 Mm glucose) supplemented with albumin from bovine serum fraction V (BSA) (Roche), 50 μM 2-mercaptoethanol, 10 mM nicotinamide (VWR), 5.5 μg/mL transferrin (Sigma-Aldrich), 6.7 ng/mL sodium selenite (Sigma-Aldrich) and 1% penicillin/streptomycin.

Cell lines were not reauthenticated during the course of the study. However, cells were checked for mycoplasma contamination regularly using MycoAlert (Lonza, LT07-318).

### Method details

#### Metabolic tests

For intraperitoneal glucose tolerance tests (IPGTT) and insulin measurements, 8–12-week-old animals were fasted for 16 h prior to experiments and received an intraperitoneal injection of glucose (1 g/kg or 3 g/kg respectively of body weight). Blood glucose levels were determined by tail venepuncture using a glucose meter (Accuchek; Roche, Burgess Hill, U). Insulin was determined by Mouse insulin ELISA (CrystalChem), according to the manufacturer’s instructions.

#### Small interfering RNA *Pax6os1* in MIN6

MIN6 cells were transfected with a pool of three small interfering RNAs (siRNAs) targeting *Pax6os1* or three control siRNAs using Lipofectamine RNAiMAX, according to the manufacturer’s protocol.

#### CRISPR-Cas9 gene editing in mice and humans and lentivirus production

Guide RNAs targeting Pax6os1/PAX6-AS1 gene in mice and humans were designed using http://crispr.mit.edu and are described in Table S2.

For the generation of Pax6os1 null mice, guide RNAs were cloned into a pX330-U6-Chimeric_BB-CBh-hSpCas9 plasmid (Addgene, #42230).^37^ Pronuclear injection of the gRNAs was performed by the MRC transgenics unit, Imperial College London. F0 compound homozygous males (carrying different mutations in the *Pax6os1* gene) were crossed with WT females C57BL/6.

For CRISPR-Cas9 in EndoC-βH1 cells, guide RNAs were cloned into lentiCRIPSR v2 (Addgene #52961),^38^ modify to expressed Cas9 under the RIP promoter. EndoC-βH1 cells were then infected with lentiviral particles that were previously generated by co-transfection of lentiCRIPR v2 together with packaging (psPAX2; Addgene, # 12260) and envelope (pMD2.G; Addgene, #12259) plasmids into HEK293T cells.

#### Transduction of pancreatic islets

Human islets were transduced as previously described^39^ with minor modifications. Briefly, islets were incubated overnight at 37°C after arrival, mildly trypsinized (3 min, 0.5X Trypsin) and infected with lentiviruses. Experiments were performed 48 h after transduction.

#### Massive parallel RNASequencing (RNASeq)

For MIN6 cells, libraries were constructed using a NEBNext Ultra II Directional RNA Library Prep Kit for Illumina (NEB) and NeBNext Multiplex Adapters (NEB) used for adapter ligation. Size selection of libraries was performed with SPRIselect Beads (Beckman Coulter). The Imperial BRC Genomics Facility performed sequencing as 75 bp paired end reads on a HiSeq4000 according to Illumina specification.

For EndoC-βH1 cells, libraries were prepared using RIBO-ZERO PLUS, following the manufacturer’s protocol. CABIMER Genomics Facility performed sequencing as 75 bp paired end reads Illumina Novaseq 6000, according to Illumina specifications.

Reads were aligned to the mouse (GRCm38) or human (GRCh38.p14) genome using HiSat2 or STAR 2.7.10^40^ and quantified featureCounts or HTSeq 2.0,^41^ respectively. Differential expression was analyzed with DESeq2^42^ and Clusterprofiler^43^ was used to determine enriched KEGG pathways.

#### Immunoblotting

The antibodies used in this study are described in key resources table. For insulin, the protocol was slightly modified, fixing the membrane with 0.2% of Glutaraldehyde followed by an antigen retrieval step prior blocking the membrane.^44^

#### Click-iT EdU (5-ethynyl-2′-deoxyuridine), cell viability and MTT assays

Control and *PAX6-AS1* null cells were fixed with 100% methanol, labeled for EdU using the Click-iT EdU Alexa Fluor 488 HCS Assay according to manufacturer’s instructions and co-stained with insulin. For cell viability, cells were cultured for 30 min in 1 mL of PBS with Calcein-AM (1 μL) (Molecular Probes) and propidium iodide (1 μL) (Sigma-Aldrich). Cells were imaged using a Nikon spinning disk microscope at ×20 magnification and counted using ImageJ software. At least 1000 cells were counted per experiment.

MTT activity was determined using the Cell Proliferation Kit I according to the recommendations of the manufacturer (Roche, Spain). Optical density was determined at 575 nm with a reference wavelength of 690 nm using a Varioskan Flash spectrophotometer (Thermo Scientific, Spain).

#### Glucose-stimulated insulin and glucagon secretion

MIN6 or EndoCB-H1 cells were incubated with KRBH buffer (120 mM NaCl, 5 mM KCl, 4 mM CaCl2, 4 mM MgCl_2_, 25 mM NaHCO_3_ and 0.2% BSA, saturated with 95% O_2_/5% CO_2_; pH 7.4) supplemented with 3- and 30-Mm glucose (MIN6) or 0.5- mM and 17- mM glucose (EndoC-βH1) for 1 h. Cells were lysed for total insulin content with 1M Tris pH 8.0, 1% Triton, 10% glycerol, 5M NaCl and 0.2 EGTA.

For insulin secretion, groups of 15 islets were incubated for 30 min in fresh KRBH buffer with 3 mM glucose at 37°C under rotation in a water bath. After mix, briefly centrifuge (700 rpm, 2 min) and removing the supernatant, islets were further incubated with 500 μl of KRBH supplemented with 17 mM glucose. For glucagon secretion, groups of 15 islets were pre-incubated in e pre-incubated in KRB solution at 5.5 mM glucose twice for 20 min followed by incubation in 1 mM glucose.^45^ Both total insulin and glucagon were extracted by adding acidified ethanol (75% ethanol/1.5% HCl). Insulin was measured by using a Homogeneous Time Resolved Fluorescence (HTRF) insulin assay kit (Cisbio) in a PHERAstar reader (BMG Labtech), following the manufacturer’s instructions. Glucagon was measured using a Glucagon ELISA kit (Mercodia) according to manufacturer’s instructions.

#### Intracellular free [Ca^2+^] measurements in islets

Groups of 15 islets were incubated with the Ca^2+^ indicator Cal-520 (Abcam, ab171868) in KRBH supplemented with 3 mM glucose for 45 min and imaged using a Zeiss Axiovert microscope equipped with a ×10 0.3–0.5 NA objective at 3mM and 17mM glucose as well as at 20 mM KCl.

#### qRT-PCR

Total RNA was extracted using TRIZOL (Invitrogen, 15596026) or PureLink RNA mini kit (Invitrogen, 12183020) and on-column PureLink DNAse (Invitrogen, 12185010) following the manufacturer’s instructions. Complementary DNA (cDNA) was synthesized using random primers (Roche) and the High-Capacity cDNA Reverse Transcription kit (Life Technologies). Real-time qPCR was performed with an SYBR Green PCR master mix (Applied Biosystems). Primers used in this study are outlined in Table S3.

#### Electrophysiology

Measurements were performed at 32°C in standard whole cell configuration using an EPC-10 amplifier and Pulse software. Exocytosis was measured using the increase of membrane capacitance in response to 500 ms depolarisations at 1 Hz. The extracellular medium was composed of: 118 NaCl mM, 5.6 KCl, mM, 2.6 CaCl mM2, 1.2 MgCl2, 5 HEPES, and 20 tetraethylammonium (TEA) (pH 7.4 with NaOH). The intracellular medium contained (in mM): 129 CsOH, 125 Glutamic acid, 20 CsCl, 15 NaCl, 1 MgCl2, 0.05 EGTA, 3 ATP, 0.1 cAMP, 5 HEPES (pH7.2 with CsOH). Cell size was estimated from the initial membrane capacitance before any stimulation. Calcium and sodium currents were measured from −60 mV to +50 mV and triggered by a 100 ms depolarisation from the resting potential (−70 mV). The effect of PAX6 loss of function on the depolarising inward current was measured at 3 independent time points: i) at the peak current occurring within the first 2ms is likely supported mainly by the rapidly inactivating voltage-gated sodium currents, ii) at 5ms of the depolarisation when the sodium component should be inactivated, iii) from 10 to 95 ms after the onset of the depolarization was used to determine the mean sustained current amplitude. The exocytosis and current measurements were normalised to the size of the cells reflecting the exocytosis and current densities expressed respectively in fF.pF^−1^ and pA.pF^−1^.

#### mRNA stability assay

EndoC-βH1 cells were treated with 5 μg/mL of Actinomycin D (Sigma-Aldrich, A9415) for 4-, 8-, 24- and 32-h prior RNA extraction.^46^ Half-life of insulin at several time points was calculated using the online software https://calculator.academy/half-life-calculator/.

#### Subcellular fractionation

MIN6 and EndoC-βH1 cells cells were lysed using 200 μL lysis buffer (10 mM NaCl, 2 mM MgCl, 10mM HEPES, 5mM dithiothreitol (DTT), 0.5% Igepal CA 630 (Sigma I3021)) and centrifuged at 8000 rpm, 4°C for 5 min. The supernatant was collected as the cytoplasmic fraction while the pellet was resuspended in 200 μL lysis buffer to yield the nuclear fraction.

#### RNA pulldown

For RNA pulldown in MIN6, *Pax6os1* or *Slc16a1* (control) sequences were cloned using Sequence- and Ligation-Independent Cloning (SLIC) into a ptRNA-S1 plasmid that harbors a T7 promoter, *tRNA-S1 (encoding the streptavidin aptamer)* and a bovine growth hormone (BGH) polyadenylation site previously available in the lab. Primers for *Pax6os1* amplification are described in Table S3. Two different SILAC MIN6 lysates were used with *Pax6os1* and *MCT1/SLC16A1* labeled with the heavy and light isotypes, respectively. Protein MIN6 lysates (4 mg) were incubated with streptavidin beads at 4°C overnight under rotation. After washes, the streptavidin aptamer and bound complex were eluted using 50 μL 10 mM Biotin (pH 7.2) (Sigma, B4501) suspended in the 1X Aptamer buffer and stored at −20°C. The pooled SILAC samples were run into an SDS-PAGE gel and cut into three slices. Each slice was subjected to in-gel tryptic digestion using a DigestPro automated digestion unit (Intavis Ltd.) and the resulting peptides were fractionated using an Ultimate 3000 nano-LC system in line with an Orbitrap Fusion Tribrid mass spectrometer (Thermo Scientific).

The RNA antisense pulldown in EndoC-βH1 cells was performed as described previously.^30^ Briefly, 100,000,000 EndoC-βH1 were UV crosslinked at 254 for 0.8 J/cm^2^.Afterward cells were lysed, sonicated and hybridized with DNA probes at 45°C for 2:30 h with 1000 rpm intermittent mixing. The equivalent of 100,000 cells per group was used for the “RNA elution sample”, while the rest was used for the “Protein elution” fraction. Probes used in this study are described in Table S4 and were purchased from IDT.

#### Secondary structure and Kmer calculations

Secondary structure for Pax6os1/PASX6-AS1 was predicted using RNAfold from the ViennaRNA^47^ package and represented using FORNA webserver (Vienna RNA web services). Kmer analysis was performed using Seekr.^48^

### Quantification and statistical analysis

For comparisons between two groups, statistical significance was calculated using non-paired two-tailed Student’s t-tests or paired Student’s t test for GSIS fold change in human islets, while repeated measurements two-way ANOVA tests were performed for metabolic tests in animals. All the statistical analyses were performed using Graph Pad Prism 8.0. or R version 4.1.1 (2021-08-10) and OriginPro2021b (OriginLab Corporation, Northampton, MA, USA). In all cases, a *p*-value <0.05 (∗) was considered statistically significant. Error bars represent the standard error of the mean (SEM). Western blot analyses were performed using ImageJ software.
