# Supplementary material for: Roles for the long non-coding RNA Pax6os1/PAX6-AS1 in pancreatic beta cell function
Source: iScience. 2024 Dec 9;28(1):111518. doi: 10.1016/j.isci.2024.111518 (PMC11731260; doi:10.1016/j.isci.2024.111518)
Supplement: Document S1. Figures S1–S6 and Tables S1–S5 [file mmc1.pdf]

iScience, Volume *28*

Supplemental information

Roles for the long non-coding RNA *Pax6os1*/*PAX6-AS1* in pancreatic beta cell function

Livia Lopez-Noriega, Rebecca Callingham, Aida Martinez-Sánchez, Sameena Nawaz, Grazia Pizza, Nejc Haberman, Nevena Cvetesic, Marie-Sophie Nguyen-Tu, Boris Lenhard, Piero Marchetti, Lorenzo Piemonti, Eelco de Koning, A.M. James Shapiro, Paul R. Johnson, Isabelle Leclerc, Benoit Hastoy, Benoit R. Gauthier, Timothy J. Pullen, and Guy A. Rutter

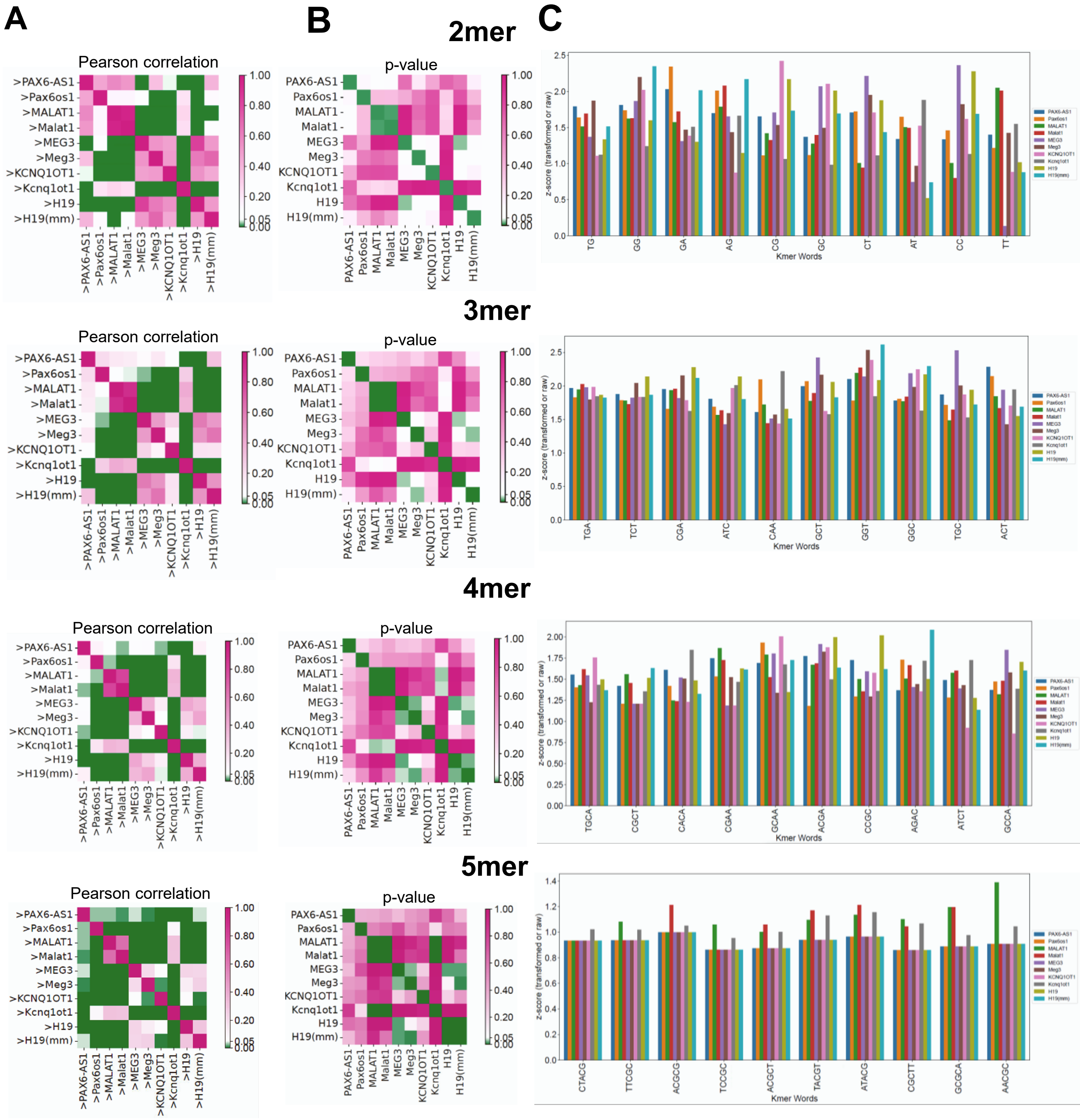

Figure S1 related to Figure 1. Correlation of different kmers between PAx6os1/PAX6AS1 and other lncRNAs with different known functions in β-cells. **A)** Heatmaps showing the pearson correlation of 2mers, 3mers, 4mers and 5mers, respectively, between mouse and human orthologs for Pax6os1/PAX6AS1, Malat1/MALAT1, Meg3/MEG3, Kcnq1ot1/KCNQ1OT1 and H19. **B)** Heatmaps depicting the p-values for the correlation found between the kmers (2-,3-,4- and 5-, respectively) for the lncRNAs described in A. **C)** Barplots showing the z-score for the most representative 2-, 3- 4- and 5-mers between the lncRNAs specified in (A) and (B).

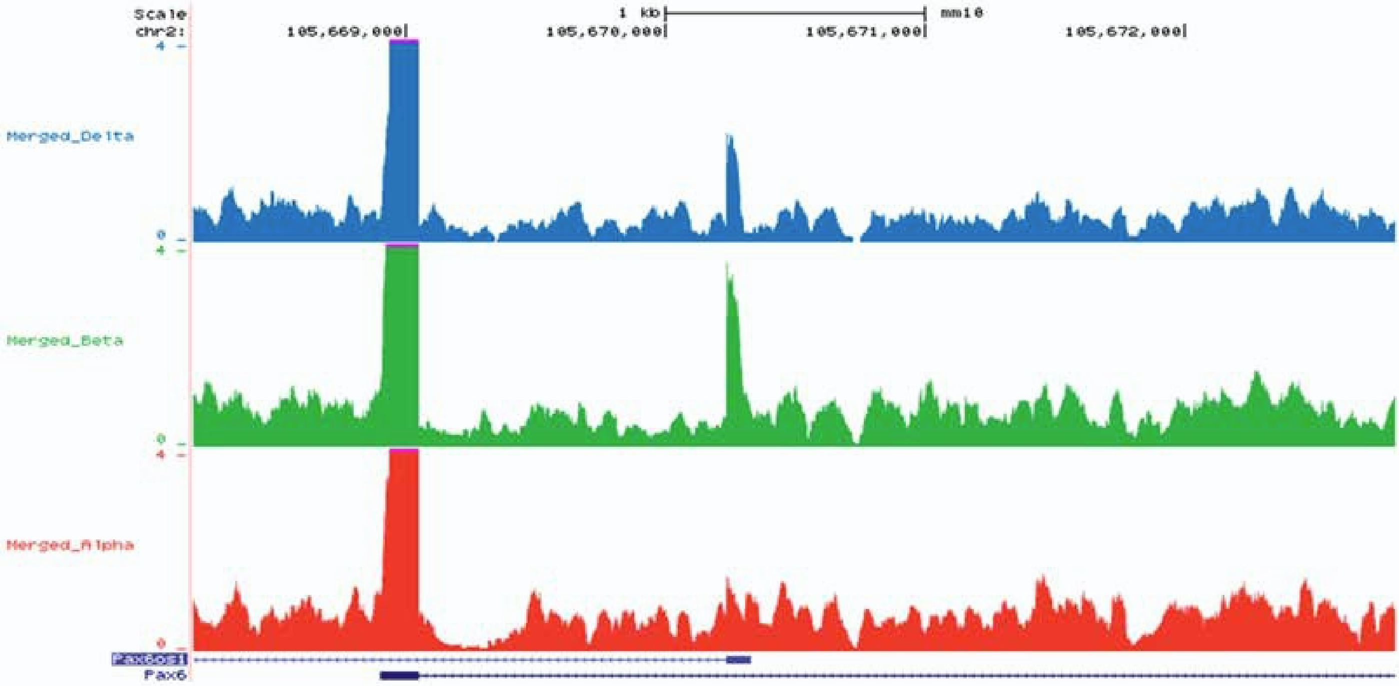

Figure S2 related to Figure 1. Pax6os1 is mainly expressed in β-cells within pancreatic islets. Genome browser tracks showing Pax6os1 expression in δ-, β- and α- cells.

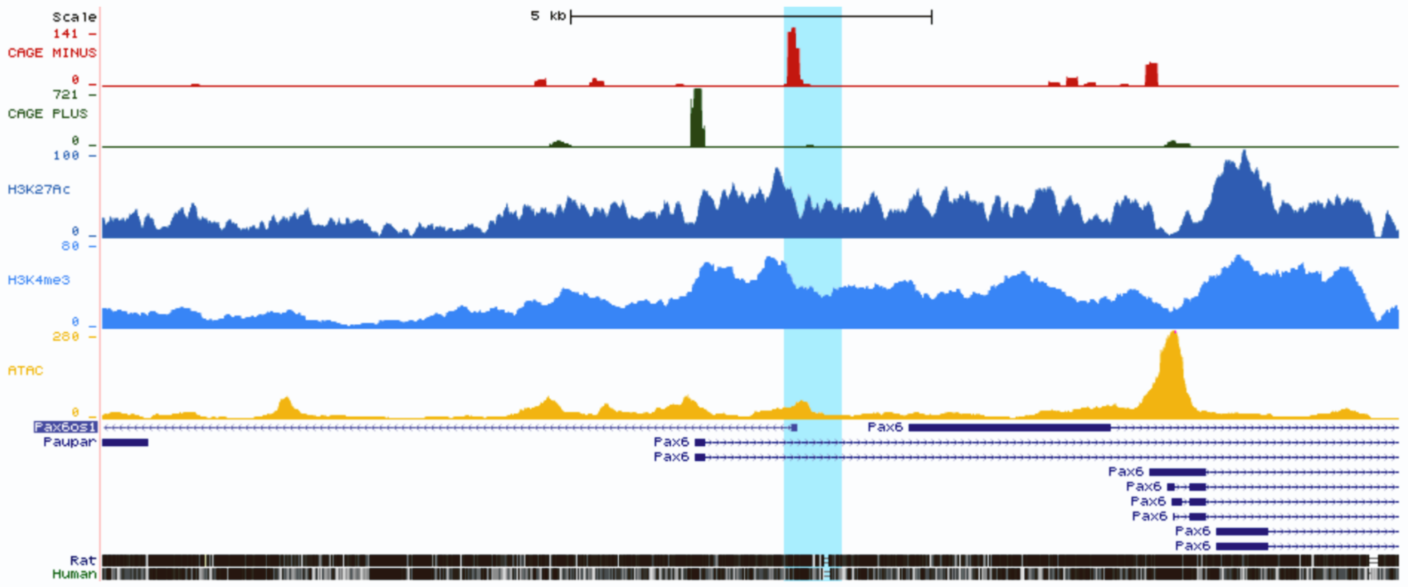

Figure S3 related to Figure 3. Chromatin landscape of the Pax6os1 locus. Integration of ATAC-seq displaying chromatin accessibility (yellow), SLIC-CAGE showing TSSs (Minus strand, red; Plus strand, green) and H3K4me3 (Lu et al., GEO: GSE110648) and H3K27Ac (Nammo et al, E-MTAB-6719) ChIP-seq datasets (blue) in mouse islets. Annotated (GENCODE VM23) of non-coding (Pax6os1 and Paupar) and coding transcripts including spliced variants (Pax6) are shown in blue, with arrows indicating transcription directionality. The region deleted in Pax6os1 KO mice is shown in pale blue. Alignment with the human DNA track is shown at the bottom.

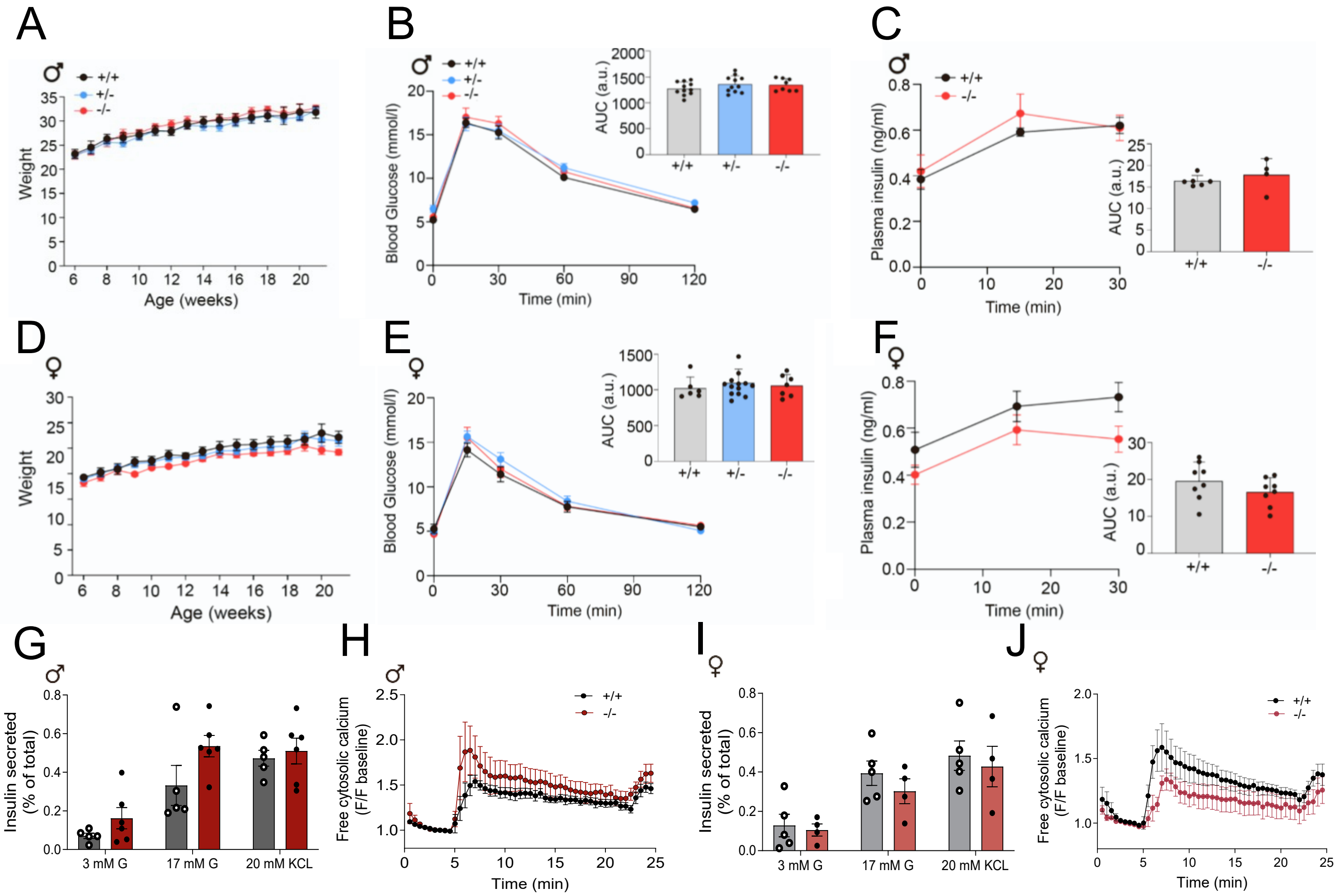

Figure S4 related to Figure 3. Pax6os1 knockout mice display normal glucose tolerance and insulin secretion compared to WT under STD. A) Body weights (g) of wt (+/+), Pax6os1heterozygous (+/-) and Pax6os1homozygous (-/-) male mice. B) Circulating glucose levels during an intraperitoneal glucose tolerance test (IPGTT). C) Plasma insulin levels after an intraperitoneal glucose load (3g/kg). D,E,F) As in panels A, B and C in female *Pax6os1* mice. G) Insulin secreted (represented as % of the total) at different glucose concentrations and after depolarization with KCl in pancreatic islets isolated from male Pax6os1 null male mice. n= 5-6. H) Intracellular calcium in pancreatic islets isolated from male Pax6os1 null male mice. n = 3. I, J) As in panels G and H but in female mice. n= 5-4 (I); n= 3 (J). Data are represented as the mean ± SEM. *p-value < 0.05 Repeated measurements two-way ANOVA

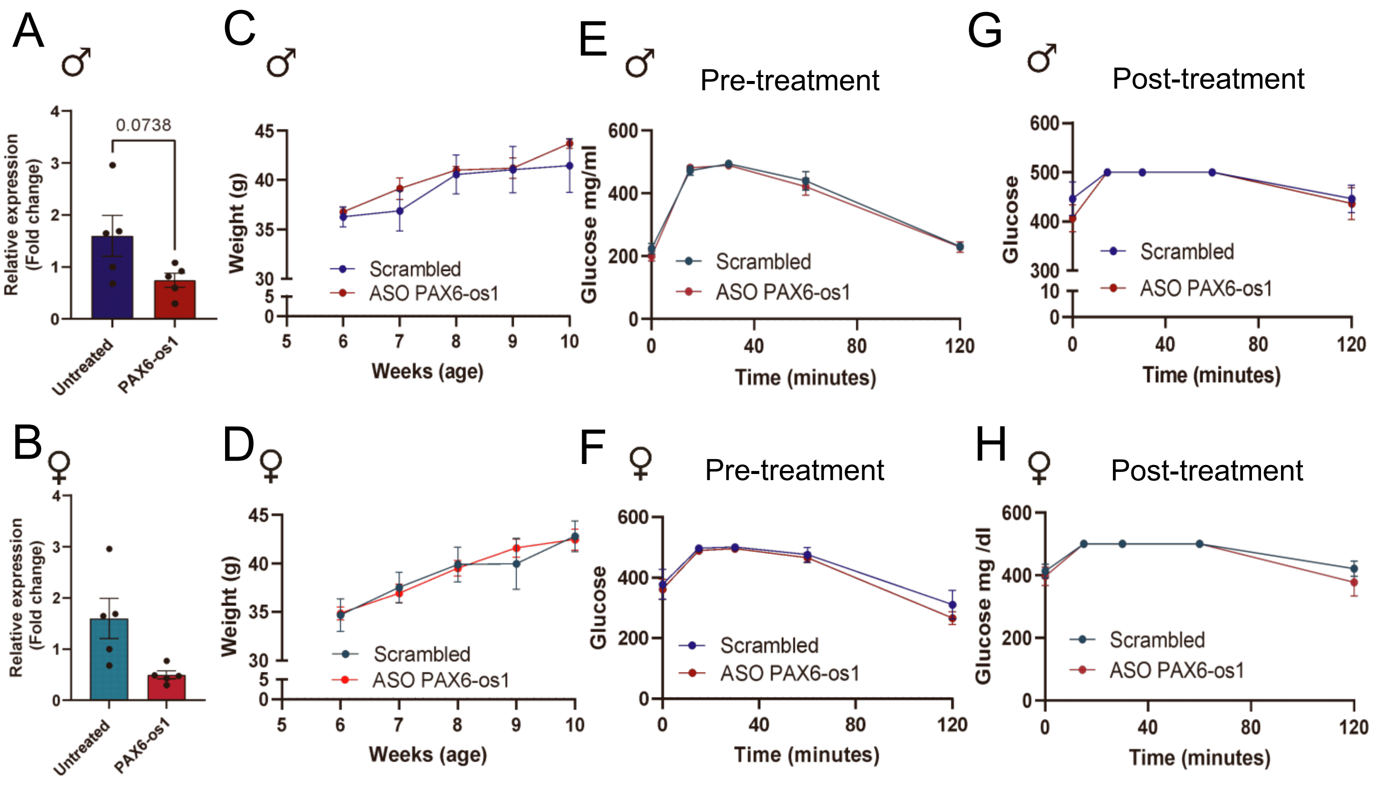

Figure S5 related to Figure 3. Db/db mice display unaltered glucose metabolism after Pax6os1 silencing using ASO. A,B) Expression of Pax6os1 in pancreatic islets isolated from db/db mice after 4 weeks of treatment with antisense oligonucleotides targeting Pax6os1. C,D) Weights (g) od db/db male and female mice for the duration of the treatment. E,F) Glucose clearance after receiving an oral glucose load (2g/kg) in male and female mice before starting the treatment. G,H) Glucose clearance of the different experimental groups at the end of the treatment. Data are repsented as the mean ± SEM. Unpaired student t test for panels A and B and two way ANOVA repeated measurements for all the other panels, *p-value<0.05.

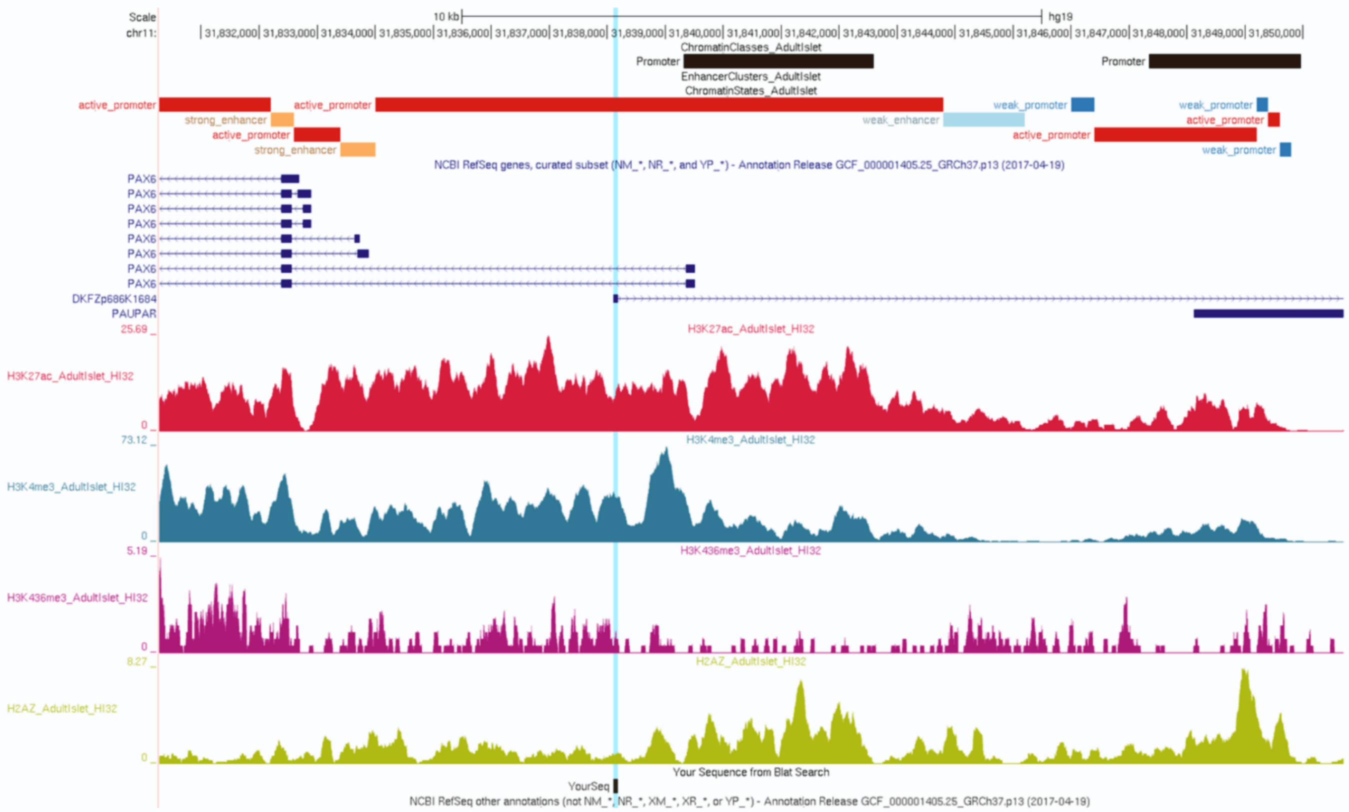

Figure S6 related to Figure 4. Diagram showing the PAX6-AS1 deleted region in human β-cells and chromatin marks found in the PAX6-AS1 and PAX6 locus (obtained from the pancreatic islet regulome browser38. PAX6-AS1 is annotated as DKFZp686K1684

Supplemental Tables

Table S1 related to Figure 1 and 6. List of donor characteristics and isolation centres.

**Identifier**	**Sex**	**Age**	**BMI**	**Isolation Centre**
49 (T2D)	Male	55	23.6	Edmonton, Canada (Macdonald)
60	Male	61	27.8	Milan
74	Male	83	24.5	Pisa
78 (T2D)	Female	54	30.8	Edmonton, Canada (McDonald)
80	Male	54	35	Edmonton, Canada (McDonald)
85	Female	62	23.9	Pisa
91 (T2D)	Female	53	21	Leiden
95	Male	38	42.6	Edmonton, Canada (McDonald)
101 (T2D)	Male	57	35	Leiden
106	Female	49	20.57	Milan
114	Female	46	35	Oxford
116	Female	55	26	Milan
127 (T2D)	Male	57	32	Oxford
165	Female	55	25	Oxford
177	Male	58	28.7	Milan
178	Female	NA	25.4	Pisa
182	Male	34	27	Oxford
188	Male	44	26	Oxford
189	Female	85	23.3	Pisa
190	Female	68	25.39	Pisa
193	Male	46	29.39	Pisa
196	Male	64	31.2	Oxford
R474	Male	48	NA	Edmonton, Canada (McDonald)
R480	Female	54	NA	Edmonton, Canada (McDonald)
R481	Female	73	NA	Edmonton, Canada (McDonald)
R485	Male	41	NA	Edmonton, Canada (McDonald)

Table S2 related to Figure 2 and 4. GuideRNAs used for CRISPR/Cas9 mediated disruption.

Human	Mouse

5’-CACCGGTCCGGCCGCACGCCTTACC-3’	CACCGTGGTGGCCACTTTGCCCG
5’-CACCGCAGGTCGCCTGCTTCGCAGT-3’	CACCGTTGTTTCCTCGGAGATCG

Table S3 related to Figure 4 and 5. Antibodies used in this study

Antibody	Dilution	Vendor	Catalog number
Anti-Pax6	1:1000 WB, 1:100 IF	Biolegend	PRB-278P
Anti-GAPDH	1:2000	Cell Signaling	2118
Anti-H4	1:1000	Cell Signaling	2935
Anti-H3	1:1000	Sigma	H0164-25UL
Goat Anti-Rabbit (HRP)	1:5000	Abcam	ab6721
Rabbit Anti-mouse (HRP)	1:5000	Sigma	A9044

Table S4 related to Figure 1, 2, 4 and 5. Primers used in this study.

Gene	Forward	Reverse
Pax6os1-202 (mouse)	AGATGCCTTAGACAAGCCTG	ATTCACCTTCTTGGACCCTG
Pax6os1-201 (mouse)	AGATGCCTTAGACAAGCCTG	ATTCACCTTCTTGGACCCTG
Pax6 (mouse)	ATGGGCGGAGTTATGATACCT	TGAAATGAGTCCTGTTGAAGTG
Beta Actin (mouse)	CGAGTCGCGTCCACCC	CATCCATGGCGAACTGGTG
Insulin 2 (mouse)	AGTAACCACCAGCCCTAAGTG	AGCACTGATCTACAATGCCAC
SLC2A2 (Glut 2) (mouse)	TTACAGTCACACCAGCATACAC	GCTTTGATCCTTCCAAGTTTGTC
Pdx1 (mouse)	GATGAAATCCACCAAAGCTC	TCGGTCAAGTTCAACATCAC

Foxa2 (mouse)	CCCATTCTGGACATGGTGAAA	AGCACGCAGAAACCATAAATTAAA
Arx (mouse)	CCGCTGGGTCTGAGCACTT	GAAAAGAGCCTGCCAAATGC
Pax4 (mouse)	ATCCAGAACCAGTCCCAAAGAG	CCAACTGGCAAACTGAAAACG
Mafa (mouse)	CAGGTGGAGCAGCTGAAGCT	CCGCCAACTTCTCGTATTTCTC
Mafb (mouse)	CGCGTCCAGCAGAAACATC	AGCTGCTCCACCTGCTGAAT
Ghrelin (mouse)	GCTGGAGATCAGGTTCAATGC	CTGCTGATACTGAGCTCCTGACA
Irx2 (mouse)	GAGGACGAAGGGATCAGTCTACA	CGGCAGGGCAATTTTTCC
Gapdh (mouse)	AGGTCGGTGTGAACGGATTTG	GGGGTCGTTGATGGCAACA
Ldha	ATGAAGGACTTGGCGGATGA	ATCTCGCCCTTGAGTTTGTCTT
RNA, U6 small nuclear 1 (RNU6-1) (mouse)	CGATACAGAGAAGATTAGCATGG	AATATGGAACGCTTCACGA
Gck (mouse)	CAACTGGACCAAGGGCTTCAA	TGTGGCCACCGTGTCATTC
PAX6-AS1 (human)	CAGCTCCAGGGAGAGGAAC	GAAGACACTCCTCCAGCAGAA
PAX6-AS1 (human)	AGCTGCTGCCTTTCTCAAAA	CATTACTGCTGAGGGCCTTG
INS (human)	GCAGCCTTTGTGAACCAACA	ACCTGCCCCACCTGCAG
Intronic INS (human)	TTGATGACCGCAGATTCAAG	CCCCATCTCCTGACTATGGA
PAX6 (human)	CCGTGTGCCTCAACCGTA	CACGGTTTACTGGGTCTGG
Cyclophilin (human)	TATCTGCACTGCCAAGACTGA	CCACAATGCTCATGCCTTCTTTCA
MAFA (human)	GCCATCGAGTACGTCAACGA	CGGGAGGCTCCTTCTTCAC
MAFB (human)	TTCTTTGGGTGAGAAGGGATCGCA	TCAGCTTGCTGCCACGTTCTCTAT

NEUROD1 (human)	ATTGCACCAGCCCTTCCTTTGATG	TCGCTGCAGGATAGTGCATGGTAA
NEUROG3 (human)	TAAGAGCGAGTTGGCACTGAGCAA	TTTGAGTCAGCGCCCAGATGTAGT
PDX1 (human)	TACTGGATTGGCGTTGTTTGTGGC	AGGGAGCCTTCCAATGTGTATGGT
SLC2A2 (human)	AGCTGCATTCAGCAATTGGACCTG	ATGTGAACAGGGTAAAGGCCAGGA
LDHA (human)	AGCCCGATTCCGTTACCT	CACCAGCAACATTCATTCCA
GHRL (human)	GGAAGATGGAGGTCAAGCAG	GCCTCTTCCCAGAGGATGTC

Table S5 related to Figure 5. DNA probes used in this study.

**PAX6-AS1.1**	[Biotin~5]GGGCAGCTGGAGAGCTGGTGCTGTGGGGAACTGCACCATTAGTCCTTCCTG
**PAX6-AS1.2**	[Biotin~5]AATGGTACAAGCATAGAGCCAAACTCTGCCCTCTGCGAGGTGCTGCTCCCA
**PAX6-AS1.3**	[Biotin~5]TTAGACTCGTAAGCCATTAAATATTAAGACTTTGTCAACGTGGCCCTGTGCACT
**PAX6-AS1.3**	[Biotin~5]CTGGGACAGACATGAACGTGTCTGTCTCTCCAGAAGATATTCAGCTTCGTGT
**Luc-1**	[Biotin~5]TCCATCCTCTAGAGGATAGAATGGCGCCGGGCCTTTCTTTATGTTTTTGGCGTCTTCCAT
**Luc-2**	[Biotin~5]CCCTTAGGTAACCCAGTAGACCCAGAGGAATTCATTATCAGTGCAATTGTTTTGTCACGA
**Luc-3**	[Biotin~5]TGTTGGGGTGTTGTAACAATATCGATTCCAATTCAGCGGGGGCCACCTGATATCCTTTGT
